# MRI factors associated with cognitive functioning after acute onset brain injury: Systematic review and meta-analysis

**DOI:** 10.1016/j.nicl.2023.103415

**Published:** 2023-04-23

**Authors:** Marlous M.L.H. Verhulst, Astrid B. Glimmerveen, Caroline M. van Heugten, Rick C.G. Helmich, Jeannette Hofmeijer

**Affiliations:** a Clinical Neurophysiology, University of Twente, Enschede, The Netherlands; b Department of Neurology, Rijnstate Hospital, Arnhem, The Netherlands; c Department of Psychiatry and Neuropsychology, School for Mental Health and Neuroscience, Maastricht University, Maastricht, The Netherlands; d Limburg Brain Injury Center, Maastricht University, Maastricht, The Netherlands; e Department of Neuropsychology and Psychopharmacology, Faculty of Psychology and Neuroscience, Maastricht University, Maastricht, The Netherlands; f Donders Institute for Brain, Cognition, and Behavior, Centre for Cognitive Neuroimaging, Radboud University Nijmegen, Nijmegen, The Netherlands; g Department of Neurology, Centre of Expertise for Parkinson & Movement Disorders, Radboud University Medical Centre, Nijmegen, The Netherlands

**Keywords:** Magnetic resonance imaging, Acute onset brain injury, Cognitive impairment, Stroke, Traumatic brain injury, Postanoxic encephalopathy

## Abstract

Impairments of memory, attention, and executive functioning are frequently reported after acute onset brain injury. MRI markers hold potential to contribute to identification of patients at risk for cognitive impairments and clarification of mechanisms. The aim of this systematic review was to summarize and value the evidence on MRI markers of memory, attention, and executive functioning after acute onset brain injury. We included ninety-eight studies, on six classes of MRI factors (location and severity of damage (n = 15), volume/atrophy (n = 36), signs of small vessel disease (n = 15), diffusion-weighted imaging measures (n = 36), resting-state functional MRI measures (n = 13), and arterial spin labeling measures (n = 1)). Three measures showed consistent results regarding their association with cognition. Smaller hippocampal volume was associated with worse memory in fourteen studies (pooled correlation 0.58 [95% CI: 0.46–0.68] for whole, 0.11 [95% CI: 0.04–0.19] for left, and 0.34 [95% CI: 0.17–0.49] for right hippocampus). Lower fractional anisotropy in cingulum and fornix was associated with worse memory in six and five studies (pooled correlation 0.20 [95% CI: 0.08–0.32] and 0.29 [95% CI: 0.20–0.37], respectively). Lower functional connectivity within the default-mode network was associated with worse cognition in four studies. In conclusion, hippocampal volume, fractional anisotropy in cingulum and fornix, and functional connectivity within the default-mode network showed consistent associations with cognitive performance in all types of acute onset brain injury. External validation and cut off values for predicting cognitive impairments are needed for clinical implementation.

## Highlights

• MRI factors in relation to cognition after acute onset brain injury were reviewed.
• Lower hippocampal volume associated with and predictive for worse cognition.
• Lower fractional anisotropy in cingulum and fornix associated with worse cognition.
• Lower connectivity in default-mode network relates to and predicts worse cognition.

## 1 Introduction

Acute onset brain injury resulting from postanoxic encephalopathy (PAE), traumatic brain injury (TBI), or stroke is a common cause of death or disability, with an incidence of 50–1,507 per 100,000 people a year, worldwide (Berdowski, 2010, Johnson, 2019, Dewan, 2018). Over the past years, survival rates have increased (Nehme, 2019, Waziry, 2020, Harrison-Felix, 2009), leading to more people living with the consequences of acute onset brain injury.

Impairments of memory, attention, and executive functioning are frequently reported in patients after acute onset brain injury (Moulaert, 2009, Lo, 2019, Rabinowitz and Levin, 2014) and strongly related to reduced quality of life (Moulaert, 2010, Cumming, 2014, Gorgoraptis, 2019). International experts recommend screening to identify patients at risk for enduring cognitive impairments (Cronberg, 2020, Winstein, 2016, Lee, 2019). Subsequently, those at risk could be provided with cognitive rehabilitation therapy.

MRI markers may contribute to identification of patients at risk for cognitive impairments and clarify underlying mechanisms. The majority of research on this topic has been performed in patients with neurodegenerative diseases or dementia. Some main findings are that lower fractional anisotropy (FA) in multiple brain regions (Mayo, 2018), lower functional connectivity (FC) within the default-mode network (DMN) (Binnewijzend, 2012), and lower hippocampal and cortical volumes (Wu, 2019) were associated with poorer cognitive performance.

In patients with acute onset brain injury similar MRI markers have been associated with cognitive performance. In ischemic stroke and TBI patients, lower hippocampal volume (Selnes, 2015), lower FA in the cingulum (De Simoni, 2016), and lower FC in resting-state networks (Palacios, 2017) were associated with poorer cognitive functioning. However, lack of uniformity of the evidence, resulting from a large variability of study designs and reported effect sizes, hampers valuing and comparing the various reported MRI markers of cognitive function after acute onset brain injury. Since patients with PAE, TBI, or stroke may face similar cognitive impairments, it is interesting to group these patients to find MRI markers associated with these impairments after acute onset brain injury. Systematic reviews, but more importantly meta-analyses summarizing and interpreting these results in the complete acute onset brain injury population are lacking. Therefore, the possible value of MRI for predicting cognitive impairments after acute onset brain injury is still unsure.

Here, we provide a systematic literature review and meta-analysis of MRI factors associated with (impairments of) memory, attention, or executive functioning after acute onset brain injury. Furthermore, we present which early MRI factors have been reported to predict long term (impairments of) memory, attention, or executive functioning after acute onset brain injury. These cognitive domains are among the most affected after stroke, TBI and PAE. With this review we aim to value and rank the evidence and provide a starting point for studies on mechanisms and predictive values of MRI factors of cognitive impairments after acute onset brain injury.

## 2 Materials and methods

The protocol was registered in the International Prospective Register for Systematic Reviews (PROSPERO:CRD42021229488) and reported here along PRISMA guidelines.

### 2.1 Search strategy

A systematic literature search was performed on January 8th 2021 using Scopus and PubMed. Search strategies are displayed in Online Resource Tables S1 and S2.

### 2.2 Selection of articles

Two reviewers (MV,AG) independently screened titles and abstracts, followed by full texts, using Rayyan QCRI. Disagreements were discussed to reach consensus.

Inclusion criteria were:

• Observational cohort studies (cross-sectional and longitudinal).
• Adult (≥18 years) acute onset brain injury population (or mixed sample with separate analysis of acute brain injury population). This included patients with TBI, stroke, and PAE. Populations with repetitive injury (sports players, soldiers or veterans, and patients with small vessel disease without acute onset injury) were excluded.
• Resting-state cerebral MRI performed (or mixed sample with separate analysis of MRI data).
• Memory, attention and/or executive functioning evaluated using neuropsychological tests with known psychometric quality and validity in the acute onset brain injury population. We only included tests with diagnostic values for distinguishing impairments from normal functioning (z-scores, norm scores, thresholds), validated in the acute onset brain injury population, and used by more than one research group. Tests primarily used and intended for research setting were not included. Cognitive impairments mostly caused by focal brain lesions, like aphasia or neglect, were not regarded.
• Published between 2000 and January 2021.
• Written in English.

### 2.3 Data extraction

The first reviewer (MV) extracted the following data from the articles: study design, inclusion & exclusion criteria, demographics (number of patients, age, sex, education), MRI (timing, sequence parameters, analysis, predictors), cognition (timing, domain, test(s), analysis, scoring method), correlation coefficients, odds ratios, group differences, or regression parameters. We assumed that MRI and cognitive testing were performed on approximately the same day in case of a cross-sectional study design.

### 2.4 Quality assessment

The first reviewer (MV) performed quality assessment based on the Quality In Prognosis Studies (QUIPS) tool (Hayden, 2013). Six bias domains were rated as having high, moderate or low risk of bias.

We used the four most important domains for our review (prognostic factor measurement, outcome measurement, study confounding, and statistical analysis and reporting) to evaluate overall risk of bias, since these domains significantly influence associations between the MRI markers and cognitive outcome measures, both in cross-sectional and longitudinal studies. Overall risk was defined as low (at least three low risk domains, no high risk domains), moderate (at least two low risk domains, at most one high risk domain), or high.

### 2.5 Data analysis

Data are presented in a descriptive way in tables and figures. The first reviewer (MV) performed meta-analyses in R (v4.0.0, R Foundation) for associations described in five or more articles (Jackson and Turner, 2017). Only correlation coefficients based on predefined ROIs were taken into account to prevent inclusion of impossibly high correlations caused by circularity errors (Vul, 2009, Vul and Pashler, 2012, Kriegeskorte, 2009). Pooled correlation coefficients were calculated with a random effects model. Missing data were requested from the corresponding authors by e-mail with a reminder after one week. In case of no or negative response, missing results were not included in meta-analyses.

## 3 Results

**Fig. 1:**
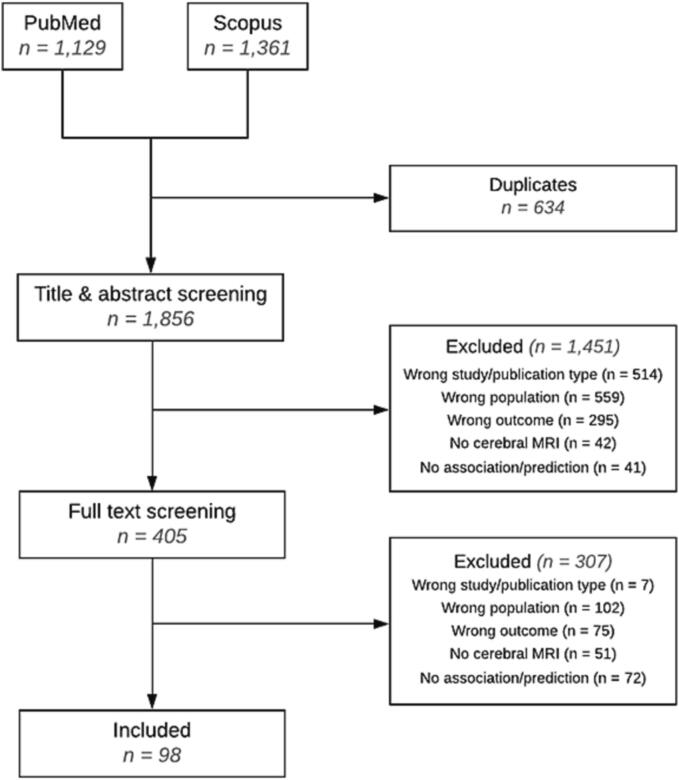
Overview of the article selection process.

Literature search resulted in 1,856 unique titles. Based on screening of title and abstract (n = 1,856) and full texts (n = 405), we included 98 articles (Fig. 1).

### 3.1 Characteristics of the included studies

The majority of studies were cross-sectional (n = 69, 70 %). The remaining 29 (30 %) were longitudinal.

All three patient populations were studied: PAE (n = 5), TBI (n = 59), and stroke (n = 34). The numbers of participants per study ranged from 5 to 395 (6,279 in total). The range of mean ages in studies on PAE was 43–64, on TBI 22–49, and on stroke 40–77 years.

Memory was tested most frequently (n = 88), followed by executive functioning (n = 59), and attention (n = 35). Used neuropsychological tests and timing of testing varied greatly. Information on used neuropsychological tests and timing is presented in Online Resource Tables S3 – S8. Six classes of MRI measures were studied: location and severity of damage (n = 15), volume/atrophy (n = 36), signs of small vessel disease (n = 15), diffusion-weighted imaging measures (n = 36), resting-state functional MRI measures (n = 13), and arterial spin labeling measures (n = 1). Time between injury and cognitive testing or MRI ranged from a few days to 49 years and time from MRI to cognitive testing from a few hours to three years.

The overall risk of bias was low in 48 studies (49 %), moderate in 39 studies (40 %), and high in eleven studies (11 %). Risk of bias was highest in study confounding and statistical analysis and reporting domains. Table 1 shows study characteristics including risk of bias.

**Table 1:** Characteristics of included studies.

First author, year of publication	Design	Population	No. of patients (no. of men)	Mean age	MRI sequence (field strength)	Cognitive domain(s)	Risk of bias
Allen, 2006	Cross-sectional	Cardiac arrest (PAE)	13 (6)	51.6	SPGR (1.5T)	Memory	Low
Arenth, 2014	Cross-sectional	Moderate/severe/complicated TBI	12 (10)	32.3	DTI (3.0T)	Memory, executive functioning	High
Ariza, 2006	Cross-sectional	TBI	20 (16)	25.0	T1 (1.5T)	Memory	Moderate
Auriat, 2019	Cross-sectional	Stroke	30 (21)	65.7	T1, T2, FLAIR (3.0T)	Memory	Low
Baek, 2013	Cross-sectional	TBI	35 (28)	36.1	DTI (1.5T)	Memory	Moderate
Bernier, 2017	Cross-sectional	Moderate to severe TBI	19 (11)	29.5	rsfMRI (NA)	Memory, executive functioning	Moderate
Chang, 2010	Cross-sectional	TBI	9 (4)	28.0	DTI (1.5T)	Memory	Moderate
Chen, 2016	Cross-sectional	Thalamic infarction	37 (28)	58.5	T1 (3.0T)	Memory	Low
Chiou, 2019	Longitudinal	Moderate to severe TBI	15 (11)	45.8	DTI (3.0T)	Executive functioning	Low
Christ et al., 2019	Longitudinal	Ischemic stroke or TIA	66 (36)	76.5	T2*, SWI (NA)	Memory, attention, executive functioning	Moderate
Chung, 2019	Cross-sectional	Mild TBI	19 (9)	30.0	DTI (3.0T)	Memory	Low
Dall'Acqua, 2017	Longitudinal	Mild TBI	49 (18)	34.9	DTI, rsfMRI (3.0T)	Memory, attention	Low
Danet, 2015	Cross-sectional	Left ischemic thalamic stroke	12 (9)	53.2	T1 (3.0T)	Memory, executive functioning	Moderate
Datta, 2009	Cross-sectional	Mild TBI	20 (15)	35.0	T1, T2, FLAIR (1.5T)	Memory, attention, executive functioning	High
De Simoni, 2016	Cross-sectional	TBI	19 (16)	38.8	DTI, rsfMRI (3.0T)	Memory, attention	Moderate
De Simoni, 2018	Cross-sectional	Moderate to severe TBI	42 (37)	40.6	DTI, rsfMRI (3.0T)	Memory, executive functioning	Low
Diao, 2017	Cross-sectional	Ischemic stroke internal capsule region	97 (72)	56.1	T1 (3.0T)	Memory	Low
Di Paola, 2008	Cross-sectional	Anoxia (PAE)	5 (5)	42.8	T1 (1.5T)	Memory, executive functioning	Moderate
Di Paola, 2015	Cross-sectional	Severe TBI	15 (14)	31.7	T1 (3.0T)	Memory, executive functioning	High
Divya, 2017	Cross-sectional	Minor stroke or TIA	50 (41)	65.0	FLAIR, SWI (NA)	Memory	Moderate
Exner et al., 2001	Cross-sectional	Thalamic infarction	15 (9)	56.0	T1 (1.5T)	Memory, attention, executive functioning	High
Fagerholm, 2015	Cross-sectional	Moderate to severe TBI	52 (35)	36.9	DTI (3.0T)	Memory, executive functioning	Low
Gale, 2005	Cross-sectional	TBI	9 (8)	29.1	T1 (1.5T)	Attention	Moderate
Geary, 2010	Cross-sectional	Mild TBI	40 (17)	34.5	DTI (3.0T)	Memory	Moderate
Gregoire, 2013	Cross-sectional	Ischemic stroke or TIA	320 (1 9 0)	63.9	T1, T2, T2*, FLAIR (1.5T)	Executive functioning	Moderate
Grossman, 2013	Longitudinal	Mild TBI	20 (16)	34.8	DKI (3.0T)	Memory, attention, executive functioning	Low
Grubb, 2000	Cross-sectional	OHCA (PAE)	17 (16)	63.9	T2, PD, GRE, FLAIR (1.0T)	Memory	Moderate
Gu, 2013	Longitudinal	TBI	15 (12)	34.8	DTI (1.5T)	Memory, attention	Moderate
Hellyer, 2013	Cross-sectional	TBI	70 (51)	35.3	DTI (3.0T)	Memory, executive functioning	Moderate
Himanen, 2005	Cross-sectional	TBI	61 (14)	29.4	T1 (1.5T)	Memory, attention	Moderate
Jang et al., 2018	Cross-sectional	TBI	86 (44)	44.5	DTI (1.5T)	Memory	Moderate
Jokinen, 2004	Cross-sectional	Ischemic stroke	260 (1 2 4)	70.1	T1, T2, PD (1.0T)	Memory, attention	Low
Jokinen, 2005	Cross-sectional	Ischemic stroke	323 (1 6 0)	70.3	T1, PD (1.0T)	Memory, attention, executive functioning	Low
Killgore, 2016	Cross-sectional	Mild TBI	26 (11)	23.4	T1 (3.0T)	Executive functioning	Low
Kinnunen, 2011	Cross-sectional	TBI	28 (21)	38.9	DTI (3.0T)	Memory, executive functioning	Low
Kondo, 2010	Cross-sectional	Severe TBI	14 (13)	24.0	DTI (1.5T)	Memory	Moderate
Kraus, 2007	Cross-sectional	TBI	37 (16)	35.4	DTI (3.0T)	Memory, attention, executive functioning	Moderate
Kuceyeski, 2011	Cross-sectional	Mild TBI	15 (10)	35.4	DTI (3.0T)	Memory	Low
Kurca et al., 2006	Cross-sectional	Mild TBI	30 (21)	32.1	T1, T2, T2*, FLAIR, DWI (1.5T)	Memory	Moderate
Lauer, 2017	Cross-sectional	TBI	39 (23)	35.2	T1 (3.0T)	Memory	Low
Leff, 2009	Cross-sectional	Stroke	210 (1 2 8)	59.0	T1 (1.5T)	Memory	Low
Liebermann, 2013	Cross-sectional	Thalamic stroke	19 (13)	44.6	T1 (1.5T)	Memory, executive functioning	High
Liu, 2017	Cross-sectional	Right hemisphere stroke	27 (20)	57.0	rsfMRI (3.0T)	Memory	Low
Liu, 2019	Longitudinal	Subcortical infarct in MCA territory	50 (30)	52.6	T1 (3.0T)	Memory	Low
Livny, 2017	Cross-sectional	TBI	24 (24)	29.0	T1 (3.0T)	Memory, executive functioning	Low
Mandzia, 2016	Longitudinal	Minor ischemic stroke or TIA	92 (68)	65.1	FLAIR, DWI (3.0T)	Memory, attention, executive functioning	Low
Martinaud, 2009	Longitudinal	AcoA aneurysm rupture	74 (35)	47.0	FLAIR, T1, T2	Memory, executive functioning	Low
Mathias, 2004	Cross-sectional	Moderate to severe TBI	25 (21)	28.0	T2, PD (1.5T)	Memory, attention, executive functioning	Moderate
Merkley, 2013	Cross-sectional	Severe TBI	12 (8)	23.0	T1 (3.0T)	Memory	Low
Muir, 2015	Cross-sectional	Ischemic stroke	106 (55)	63.8	T1, FLAIR, DWI (1.5/3.0T)	Executive functioning	Low
Munir, 2019	Longitudinal	Minor ischemic stroke or TIA	80 (63)	64.5	T1 (3.0T)	Memory, executive functioning	Low
Munivenkatappa, 2016	Longitudinal	Mild TBI	21 (NA)	26.3	DTI (NA)	Memory, attention, executive functioning	High
Munivenkatappa, 2017	Longitudinal	Mild TBI	21 (NA)	26.3	DTI (NA)	Memory, attention, executive functioning	High
Munivenkatappa, 2019	Longitudinal	Mild TBI	21 (NA)	26.3	T1 (NA)	Memory, attention, executive functioning	High
Orbo, 2018	Cross-sectional	OHCA (PAE)	26 (25)	58.6	T1 (1.5T)	Memory	Moderate
Ostberg, 2020	Longitudinal	TBI	114 (72)	49.0	T1 (3.0T)	Attention	Moderate
Owens, 2017	Cross-sectional	Moderate to severe TBI	20 (15)	39.1	DTI (3.0T)	Memory, attention	Low
Palacios, 2011	Cross-sectional	Severe TBI	15 (NA)	23.6	DTI (1.5T)	Memory	Low
Palacios, 2013b	Cross-sectional	Severe TBI	26 (16)	27.4	T1, DTI (3.0T)	Memory	Low
Palacios, 2013a	Cross-sectional	TBI	26 (16)	27.4	T1, DTI (3.0T)	Memory, attention	Moderate
Palacios, 2017	Longitudinal	Mild TBI	75 (50)	32.3	rsfMRI (3.0T)	Memory, attention	High
Peng, 2016	Cross-sectional	Subcortical stroke	32 (22)	59.6	rsfMRI (3.0T)	Memory, attention, executive functioning	Low
Pohjasvaara, 2007	Cross-sectional	Ischemic stroke	395 (1 9 1)	70.5	PD (1.0T)	Memory, executive functioning	Low
Rabinowitz, 2018	Longitudinal	Moderate to severe TBI	46 (32)	35.2	DTI (3.0T)	Memory, executive functioning	Moderate
Rajagopalan, 2019	Cross-sectional	Moderate to severe TBI	17 (12)	27.7	T1, DTI (3.0T)	Memory, executive functioning	Low
Sachdev, 2007a	Longitudinal	Stroke or TIA	47 (29)	72.0	T1 (1.5T)	Memory	Low
Sachdev, 2007b	Cross-sectional	Stroke or TIA	90 (57)	72.1	T1, FLAIR (1.5T)	Memory, attention, executive functioning	Moderate
Sachdev, 2014	Longitudinal	Ischemic stroke or TIA	183 (1 0 9)	71.9	T1, FLAIR (1.5T)	Memory, attention, executive functioning	Moderate
Santhanam, 2019	Cross-sectional	Mild TBI	51 (50)	32.4	rsfMRI (3.0T)	Memory	Moderate
Schaapsmeerders, 2015b	Cross-sectional	Ischemic stroke	176 (78)	39.6	T1 (1.5T)	Memory	Low
Schaapsmeerders, 2015a	Cross-sectional	Ischemic stroke	146 (67)	39.6	DTI (1.5T)	Memory	Low
Schouten, 2009	Longitudinal	Supratentorial nonlacunar infarction	86 (42)	63.3	T1, T2, FLAIR (0.5/1.0/1.5T)	Memory	Low
Selnes, 2015	Cross-sectional	Cortical and lacunar infarction	27 (21)	63.7	T1, FLAIR (1.5T)	Memory, executive functioning	Low
Shah, 2012	Cross-sectional	Severe TBI	14 (12)	22.0	T1, DTI (3.0T)	Executive functioning	Moderate
Shumskaya, 2017	Cross-sectional	TBI	43 (25)	42.3	rsfMRI (3.0T)	Memory, attention, executive functioning	Moderate
Solmaz, 2017	Cross-sectional	Moderate to severe TBI	40 (NA)	NA	DTI (3.0T)	Memory, executive functioning	High
Spitz, 2013b	Cross-sectional	TBI	79 (64)	41.4	FLAIR, SWI (3.0T)	Memory, attention, executive functioning	Moderate
Spitz, 2013c	Cross-sectional	TBI	69 (56)	34.7	T1 (3.0T)	Memory, attention, executive functioning	Moderate
Spitz, 2013a	Cross-sectional	TBI	78 (63)	38.6	DTI (3.0T)	Memory, attention, executive functioning	Moderate
Stamenova, 2018	Cross-sectional	Brief OHCA (PAE)	9 (7)	55.0	SPGR (3.0T)	Memory, executive functioning	High
Stewan Feltrin, 2018	Longitudinal	TBI	24 (20)	28.0	T1, SWI (3.0T)	Memory, attention	Moderate
Sugiyama, 2009	Cross-sectional	TBI	11 (10)	35.4	DTI (1.5T)	Memory, attention, executive functioning	Low
Tang, 2011	Longitudinal	Acute ischemic stroke	328 (1 4 7)	71.4	T1, FLAIR, GRE, DWI (1.5T)	Memory, attention, executive functioning	Low
Tuladhar, 2013	Cross-sectional	Ischemic stroke	20 (13)	55.1	rsfMRI (1.5T)	Memory	Low
Van der Horn, 2017	Longitudinal	Mild TBI	53 (35)	33.4	DTI (3.0T)	Memory	Low
Vannorsdall, 2010	Cross-sectional	TBI	14 (10)	43.5	SPGR (1.5T)	Memory, executive functioning	Moderate
Van Rooij, 2017	Longitudinal	TIA or TNA	121 (74)	64.6	T1, T2, T2*, FLAIR, DWI (1.5T)	Memory, executive functioning	Moderate
Vataja, 2003	Cross-sectional	Ischemic stroke	214 (1 0 4)	69.9	T1, PD (1.0T)	Executive functioning	Moderate
Wallace, 2020	Cross-sectional	TBI	165 (1 3 0)	43.6	DTI (3.0T)	Memory, executive functioning	Low
Ware, 2020	Longitudinal	Moderate to severe TBI	36 (26)	33.8	T1, ASL (3.0T)	Memory, executive functioning	Low
Wright, 2013	Longitudinal	TBI	14 (11)	28.9	T1 (1.5T)	Memory, attention, executive functioning	Low
Xiong, 2016	Cross-sectional	Mild TBI	25 (16)	32.5	rsfMRI (3.0T)	Memory	Moderate
Xu, 2018	Longitudinal	Mild TBI	50 (30)	37.2	rsfMRI (3.0T)	Memory	Low
Yamagata, 2020	Cross-sectional	TBI	18 (NA)	42.1	DTI (3.0T)	Attention	Moderate
Yao, 2020	Cross-sectional	Stroke with basal ganglia damage	14 (8)	61.0	rsfMRI (3.0T)	Memory	Low
Yatawara, 2020	Longitudinal	Ischemic stroke	346 (2 0 8)	61.8	T1, T2, DWI (1.5T)	Attention, executive functioning	Low
Yoo, 2014	Cross-sectional	TBI	20 (16)	33.9	DTI (1.5T)	Memory	Low
Zuo, 2018	Cross-sectional	Mild stroke with basal ganglia infarcts	33 (27)	50.7	DTI (3.0T)	Memory, executive functioning	Low

AcoA = anterior communicating artery, ASL = arterial spin labeling, DKI = diffusion kurtosis imaging, DTI = diffusion tensor imaging, DWI = diffusion-weighted imaging, FLAIR = fluid attenuated inversion recovery, GRE = gradient echo, MCA = middle cerebral artery, NA = not available, OHCA = out-of-hospital cardiac arrest, PAE = postanoxic encephalopathy, PD = proton density, rsfMRI = resting-state functional MRI, SPGR = spoiled gradient echo, SWI = susceptibility-weighted imaging, TBI = traumatic brain injury, TIA = transient ischemic attack, TNA = transient neurological attack.

### 3.2 Results of the included studies

#### 3.2.1 Location and extent of damage

Characteristics and results of studies on location and extent of damage are summarized in Online Resource Table S3. This class was only investigated after TBI or stroke.

##### 3.2.1.1 Associations

Larger lesion volume (Danet, 2015, Exner et al., 2001, Spitz, 2013b), damage to the mammillothalamic tract (Danet, 2015), lower grey matter density (Leff, 2009), and lower grey matter/white matter contrast (Palacios, 2013a) were associated with worse memory performance. Six studies reported absence of an association between damage and memory (Datta, 2009, Diao, 2017, Liebermann, 2013, Liu, 2019, Martinaud, 2009, van Rooij, 2017).

Left-sided lesions were associated with worse attention performance than right-sided lesions (Exner et al., 2001). Three studies reported absence of an association between damage and attention (Spitz, 2013b, Palacios, 2013a, Datta, 2009).

Damage to the lateral cholinergic projections (Muir, 2015) was associated with worse executive functioning. Four studies reported absence of an association between damage and executive functioning (Danet, 2015, Exner et al., 2001, Palacios, 2013a, Datta, 2009).

##### 3.2.1.2 Prediction

Larger lesion volume was predictive for worse memory performance at one year, while right sided lesions and cortical lesions were predictive for better memory performance (Schouten, 2009).

Bilateral lesions were predictive for worse executive functioning (Mandzia, 2016). Presence of DWI lesions was predictive for more decline in executive functioning in the first six months (van Rooij, 2017).

#### 3.2.2 Volume/atrophy

Characteristics and results of studies on cerebral volume/atrophy are summarized in Online Resource Table S4.

##### 3.2.2.1 Associations

**Fig. 2:**
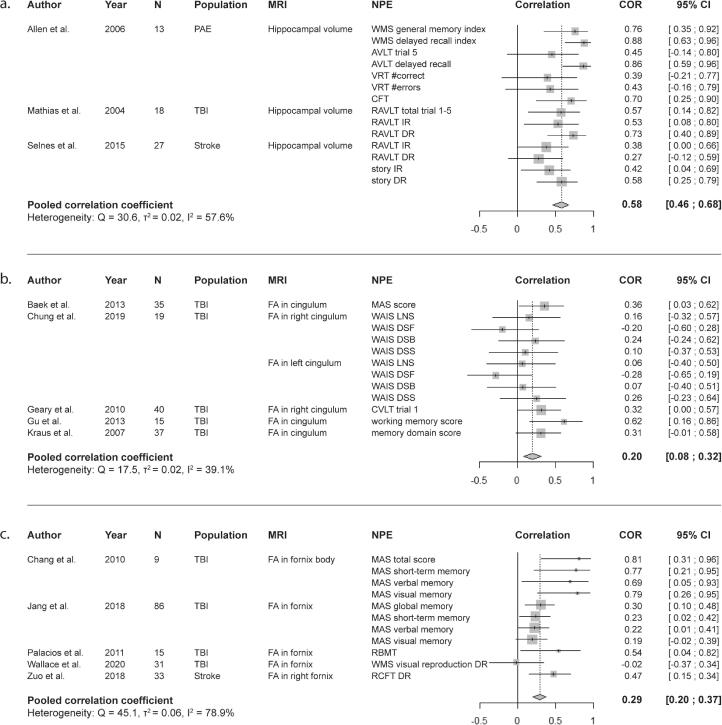
Forest plots for the correlations between MRI markers and memory functioning. a. Correlation between total hippocampal volume and memory functioning. b. Correlation between fractional anisotropy in the cingulum and memory functioning. c. Correlation between fractional anisotropy in the fornix and memory functioning. AVLT = Auditory Verbal Learning Test, CFT = Complex Figure Test, CI = confidence interval, COR = correlation coefficient, CVLT = California Verbal Learning Test, DR = delayed recall, DSB = Digit Span Backward, DSF = Digit Span Forward, DSS = Digit Span Sequencing, FA = fractional anisotropy, IR = immediate recall, LNS = Letter Number Sequencing, MAS = Memory Assessment Scale, N = number of patients, NPE = neuropsychological examination, PAE = postanoxic encephalopathy, RAVLT = Rey Auditory Verbal Learning Test, RBMT = Rivermead Behavioural Memory Test, RCFT = Rey Complex Figure Test, TBI = traumatic brain injury, VRT = Visual Retention Test, WAIS = Wechsler Adult Intelligence Scale, WMS = Wechsler Memory Scale.

**Fig. 3:**
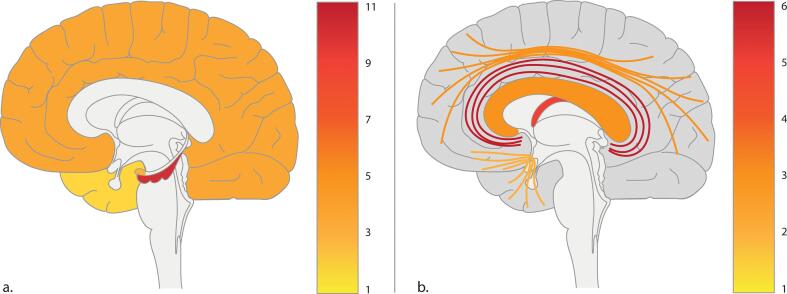
Number of studies that found a significant positive correlation between MRI markers and memory performance. a. Positive correlation between brain volume and memory performance. Highlighted structures include (dark to light) hippocampus, cortex, amygdala, and temporal lobe. b. Positive correlation between fractional anisotropy and memory performance. Highlighted regions include (dark to light) cingulum, fornix, corpus callosum, superior longitudinal fasciculus, and uncinate fasciculus.

Studies in all three populations showed significant associations between volumes of hippocampus or amygdala and memory performance, wherein poorer memory performance was associated with lower volumes (Selnes, 2015, Allen, 2006, Grubb, 2000, Orbo, 2018, Ariza, 2006, Di Paola, 2015, Lauer, 2017, Mathias, 2004, Chen, 2016, Sachdev, 2007a, Sachdev, 2007b, Stamenova, 2018, Himanen, 2005, Schaapsmeerders, 2015b). Only one study showed absence of a significant correlation (Di Paola, 2008). Pooled correlation coefficients for whole, left and right hippocampus with memory performance were 0.58 [95% CI: 0.46–0.68], 0.11 [95% CI: 0.04–0.19], and 0.34 [95% CI: 0.17–0.49], respectively (Fig. 2a, Table S1). Demographic data and study design of the studies included in meta-analyses are shown in Table 2. Furthermore, smaller volumes of grey matter (Diao, 2017, Allen, 2006, Orbo, 2018, Lauer, 2017, Palacios, 2013b, Spitz, 2013c), white matter (Allen, 2006, Lauer, 2017, Vannorsdall, 2010), multiple brain lobes (Grubb, 2000, Munivenkatappa, 2019), cingulate gyrus (Munivenkatappa, 2019), and mammillothalamic tract (Danet, 2015) were correlated with worse memory performance. On the other hand, larger volumes of CSF (Lauer, 2017, Himanen, 2005, Auriat, 2019) and higher atrophy rates of cortex (Jokinen, 2005), left thalamus (Liu, 2019), and medial temporal lobe (Jokinen, 2004) were associated with worse performance. Five studies reported absence of an association between brain volume and memory (Livny, 2017, Merkley, 2013, Ware, 2020, Munir, 2019, Stewan Feltrin, 2018). Fig. 3a shows an overview of the number of studies that described a positive correlation between brain volumes and memory performance.

**Table 2:** Demographics of the studies included in meta-analysis.

First author, year of publication	Design	Population	No. of patients (no. of men)	Mean age	MRI sequence (field strength)	Timing of MRI	Timing of cognitive testing
Volume of hippocampus							
Allen, 2006	Cross-sectional	Cardiac arrest (PAE)	13 (6)	51.6	SPGR (1.5T)	6M – 10Y	6M – 10Y
Mathias, 2004	Cross-sectional	Moderate to severe TBI	25 (21)	28.0	T2, PD (1.5T)	± 213D	± 213D
Selnes, 2015	Cross-sectional	Cortical and lacunar infarction	27 (21)	63.7	T1, FLAIR (1.5T)	3M	3M

Volume of left hippocampus							
Grubb, 2000	Cross-sectional	OHCA (PAE)	17 (16)	63.9	T2, PD, GRE, FLAIR (1.0T)	6M – 22M	6M – 22 M
Di Paola, 2015	Cross-sectional	Severe TBI	15 (14)	31.7	T1 (3.0T)	60D – 887D	60D – 887D
Sachdev, 2007b	Cross-sectional	Stroke or TIA	90 (57)	72.1	T1, FLAIR (1.5T)	3M – 6M	3M – 6M
Stamenova, 2018	Cross-sectional	Brief OHCA (PAE)	9 (7)	55.0	SPGR (3.0T)	> 5M	> 5M

Volume of right hippocampus							
Grubb, 2000	Cross-sectional	OHCA (PAE)	17 (16)	63.9	T2, PD, GRE, FLAIR (1.0T)	6M – 22M	6M – 22M
Sachdev, 2007b	Cross-sectional	Stroke or TIA	90 (57)	72.1	T1, FLAIR (1.5T)	3M – 6M	3M – 6M
Stamenova, 2018	Cross-sectional	Brief OHCA (PAE)	9 (7)	55.0	SPGR (3.0T)	> 5M	> 5M

FA in cingulum							
Baek, 2013	Cross-sectional	TBI	35 (28)	36.1	DTI (1.5T)	99D – 495D	100D – 502D
Chung, 2019	Cross-sectional	Mild TBI	19 (9)	30.0	DTI (3.0T)	± 16D	± 16D
Geary, 2010	Cross-sectional	Mild TBI	40 (17)	34.5	DTI (3.0T)	> 6M	> 6M
Gu, 2013	Longitudinal	TBI	15 (12)	34.8	DTI (1.5T)	< 7D	12M – 26M
Kraus, 2007	Cross-sectional	TBI	37 (16)	35.4	DTI (3.0T)	> 6M	> 6M

FA in fornix							
Chang, 2010	Cross-sectional	TBI	9 (4)	28.0	DTI (1.5T)	53D – 350D	53D – 350D
Jang et al., 2018	Cross-sectional	TBI	86 (44)	44.5	DTI (1.5T)	± 6M	± 6M
Palacios, 2011	Cross-sectional	Severe TBI	15 (NA)	23.6	DTI (1.5T)	86D – 660D	86D – 660D
Wallace, 2020	Cross-sectional	TBI	165 (1 3 0)	43.6	DTI (3.0T)	± 200D	± 200D
Zuo, 2018	Cross-sectional	Mild stroke with basal ganglia infarcts	33 (27)	50.7	DTI (3.0T)	10D – 14D	< 10D

D = days, DTI = diffusion tensor imaging, FLAIR = fluid attenuated inversion recovery, GRE = gradient echo, M = months, OHCA = out-of-hospital cardiac arrest, PAE = postanoxic encephalopathy, PD = proton density, SPGR = spoiled gradient echo, TBI = traumatic brain injury, TIA = transient ischemic attack, Y = years.

Smaller volumes of grey matter (Gale, 2005) and cingulate gyrus (Munivenkatappa, 2019), and higher atrophy rates of cortex (Jokinen, 2005, Ostberg, 2020), white matter (Stewan Feltrin, 2018), multiple gyri (Ostberg, 2020), and temporal lobe (Jokinen, 2004) were associated with worse attention performance. Five studies reported absence of an association between brain volume and attention (Mathias, 2004, Sachdev, 2007b, Himanen, 2005, Spitz, 2013b, Sachdev, 2014).

One study showed a significant association between lower hippocampal volume and worse executive functioning (Himanen, 2005), while three other studies showed no association (Selnes, 2015, Di Paola, 2015, Sachdev, 2014). Additionally, smaller cortical volumes (Spitz, 2013c, Livny, 2017, Killgore, 2016), larger ventricle volumes (Himanen, 2005, Ware, 2020), and higher atrophy rates of whole brain (Stewan Feltrin, 2018), temporal and frontal lobes (Wright, 2013, Vataja, 2003), and cortex (Jokinen, 2005) were associated with worse performance. Eight studies reported absence of an association between brain volume and executive functioning (Danet, 2015, Mathias, 2004, Sachdev, 2007a, Stamenova, 2018, Di Paola, 2008, Vannorsdall, 2010, Munivenkatappa, 2019, Munir, 2019).

##### 3.2.2.2 Prediction

Lower hippocampal volume at three to six months was predictive for memory decline between three to six months and three years (Sachdev, 2014). Furthermore, higher atrophy rates of total brain, right and left temporal lobe, and frontal lobe were predictive for attention and executive functioning, but not memory performance, at twelve months (Wright, 2013).

#### 3.2.3 Signs of small vessel disease

Characteristics and results of studies on small vessel disease measures are summarized in Online Resource Table S5. This class was only investigated after stroke.

##### 3.2.3.1 Associations

More severe white matter hyperintensities (WMHs) (Pohjasvaara, 2007) and presence of WMHs in target regions (Jokinen, 2005), were associated with worse memory performance. Four studies reported absence of an association between WMHs or cerebral microbleeds (CMBs) and memory (Selnes, 2015, Exner et al., 2001, van Rooij, 2017, Auriat, 2019).

WMHs in target regions were associated with worse attention performance (Jokinen, 2005). Three studies reported absence of an association between WMHs or CMBs and attention (Exner et al., 2001, Sachdev, 2014, Yatawara, 2020).

More severe WMHs (Selnes, 2015, Muir, 2015, Vataja, 2003, Pohjasvaara, 2007), WMHs in target regions (Jokinen, 2005, Vataja, 2003), and number and location of CMBs (Gregoire, 2013) were associated with worse executive functioning. Four studies reported absence of an association between WMHs or CMBs and executive functioning (Exner et al., 2001, van Rooij, 2017, Sachdev, 2014, Yatawara, 2020).

##### 3.2.3.2 Prediction

WMH volume was predictive for larger memory decline in the first three years after the event (Sachdev, 2014). Furthermore, presence of CMBs, multilocular CMBs, more WMHs, and more deep WMHs were predictive for worse memory performance at three to six months (Christ et al., 2019, Divya, 2017). One study showed no prediction of reverting from memory impairments to normal memory functioning based on CMBs (Tang, 2011).

Presence of CMBs and multilocular CMBs were also predictive for worse executive functioning at six months, but not for attention performance (Christ et al., 2019).

#### 3.2.4 Diffusion-weighted imaging

Characteristics and results of studies on diffusion-weighted imaging measures are summarized in Online Resource Table S6. This class was only investigated after TBI or stroke.

##### 3.2.4.1 Associations

Lower FA in corpus callosum (Arenth, 2014, Geary, 2010, Palacios, 2011), cingulum (De Simoni, 2016, Palacios, 2013b, Geary, 2010, Baek, 2013, Sugiyama, 2009), fornix (Palacios, 2011, Chang, 2010, Jang et al., 2018, Kinnunen, 2011, Zuo, 2018), uncinate fasciculus (Geary, 2010), and superior longitudinal fasciculus (Palacios, 2013b, Geary, 2010) were associated with worse memory performance. A larger number of brain regions with reduced FA correlated with worse functioning (Kraus, 2007). Pooled correlation coefficients for FA in cingulum and fornix with memory performance were 0.20 [95% CI: 0.08–0.32] and 0.29 [95% CI: 0.20–0.37], respectively (Fig. 2b and c). Demographic data and study design of the studies included in meta-analyses are shown in Table 2. Fig. 3b shows an overview of the number of studies that described a positive correlation between FA of specific brain regions and memory performance.

Results for diffusivity measures were not as straightforward. Worse memory performance was associated with lower diffusivity in thalamus (Munivenkatappa, 2016, Munivenkatappa, 2017), external capsule (Grossman, 2013), corpus callosum (Grossman, 2013), and total white matter (Grossman, 2013), and higher diffusivity in splenium (Arenth, 2014) and temporal lobe (Munivenkatappa, 2017). Higher kurtosis in cingulum, centrum semiovale, and total white matter was associated with worse performance (Grossman, 2013), while lower kurtosis in superior longitudinal fasciculus was associated with worse memory (Chung, 2019).

In structural connectivity analyses, disruptions of the cingulum (Baek, 2013), fornix (Jang et al., 2018), connection between cingulum and brainstem (Yoo, 2014), or structural connectome (Solmaz, 2017) were found in patients with worse memory performance. Higher betweenness centrality in multiple gyri was associated with better memory functioning (Fagerholm, 2015). Seven studies reported absence of an association between anisotropy or diffusivity and memory (De Simoni, 2018, Hellyer, 2013, Owens, 2017, Rajagopalan, 2019, Spitz, 2013a, Wallace, 2020, Schaapsmeerders, 2015a).

Lower FA in corpus callosum (Spitz, 2013a), cingulum (Sugiyama, 2009), and superior longitudinal fasciculus (Spitz, 2013a) was associated with worse attention functioning. Higher diffusivity in the thalamus was associated with worse attention functioning (Munivenkatappa, 2016, Munivenkatappa, 2017). Lower kurtosis was associated with worse attention performance (Grossman, 2013). Four studies reported absence of an association between anisotropy or diffusivity and attention (De Simoni, 2016, Palacios, 2013a, Zuo, 2018, Owens, 2017).

Lower FA in corpus callosum (Arenth, 2014), cingulum (Chiou, 2019), uncinate fasciculus (Chiou, 2019), and superior longitudinal fasciculus (Spitz, 2013a, Chiou, 2019) was also associated with worse executive functioning. A higher number of brain regions with reduced FA was associated with worse performance (Kraus, 2007). Higher diffusivity in corpus callosum (Arenth, 2014, Hellyer, 2013), cingulum (Hellyer, 2013), and frontal white matter (Kinnunen, 2011, Munivenkatappa, 2017) was associated with worse executive functioning, as well. In structural connectivity analysis, disruption of structural connectome was associated with worse executive functioning (Solmaz, 2017). Lower eigenvector centrality in left peri-callosal region, right superior frontal gyrus, left thalamus, left caudate, left insula, and right cingulate cortex was associated with worse executive functioning (Fagerholm, 2015). Four studies reported absence of an association between anisotropy or diffusivity and executive functioning (Zuo, 2018, Munivenkatappa, 2016, Grossman, 2013, Wallace, 2020).

##### 3.2.4.2 Prediction

Lower FA and higher diffusivity in cingulum, superior longitudinal fasciculus, and interior longitudinal fasciculus was predictive for worse memory performance at least one year later (Gu, 2013). However, in the uncinate fasciculus, both lower FA and lower AD were predictive for worse memory performance (Gu, 2013). Furthermore, higher betweenness centrality in the left inferior frontal gyrus and left superior temporal gyrus was predictive for worse memory performance after three months (van der Horn, 2017).

In contrast, higher FA and lower diffusivity in anterior corona radiate, superior longitudinal fasciculus, and inferior longitudinal fasciculus were predictive for worse attention performance at least one year later (Gu, 2013).

#### 3.2.5 Resting-state functional MRI

Characteristics and results of studies on resting-state functional MRI markers are summarized in Online Resource Table S7. This class was only investigated after TBI or stroke.

##### 3.2.5.1 Associations

Lower FC between nodes within the DMN (De Simoni, 2016, Santhanam, 2019, Dall'Acqua, 2017) and between the DMN and right middle frontal gyrus (Liu, 2017) was associated with worse memory performance. In contrast, higher FC between DMN and right cingulate gyrus, left claustrum, and left inferior frontal gyrus was associated with worse performance (Liu, 2017). Additionally, multiple functional connections were associated with memory functioning: FC within right anterior caudate (De Simoni, 2018), FC between left thalamus and left middle frontal gyrus (Xiong, 2016), FC between dorsal attention network and right precentral gyrus (Liu, 2017), and FC between bilateral temporal gyri (Yao, 2020). Decreased spontaneous neural activity and decreased regional homogeneity in the cingulate gyrus (Xiong, 2016, Peng, 2016) were associated with worse memory functioning. Four studies reported absence of an association between FC and memory (Bernier, 2017, Shumskaya, 2017, Xu, 2018, Tuladhar, 2013).

Lower FC within the DMN (De Simoni, 2016, Palacios, 2017, Dall'Acqua, 2017) and sensorimotor network (Shumskaya, 2017) was associated with worse attention functioning. Furthermore, decreased regional homogeneity within the cingulate cortex was associated with worse attention (Peng, 2016).

Lower FC in caudate (De Simoni, 2018) was associated with worse executive functioning. Three studies reported absence of an association between FC and executive functioning (Peng, 2016, Bernier, 2017, Shumskaya, 2017).

##### 3.2.5.2 Prediction

Lower FC in the occipital cerebellar network was predictive for worse memory performance six months later (Palacios, 2017).

Lower FC in the DMN, salience network, dorsal attention network, and orbitofrontal network was predictive for worse attention six months later (Palacios, 2017). Furthermore, higher FC between the basal ganglia and orbitofrontal network was predictive for worse attention performance (Palacios, 2017).

#### 3.2.6 Arterial spin labeling

Characteristics and results of studies on arterial spin labeling measures are summarized in Online Resource Table S8. This class was only investigated after TBI.

##### 3.2.6.1 Associations

Decreased cerebral blood flow in regions of interest (regions with lower cerebral blood flow in patients than in healthy controls) was associated with worse executive functioning (Ware, 2020).

##### 3.2.6.2 Prediction

Decreased cerebral blood flow in regions of interest was not predictive for memory or executive functioning at six to twelve months (Ware, 2020).

## 4 Discussion

With this systematic review and meta-analysis we provide an overview of MRI factors associated with or predictive for (impairments of) memory, attention, or executive functioning after acute onset brain injury. Our results show that hippocampal volume, fractional anisotropy in multiple regions, and functional connectivity within the default-mode network were associated with in cross-sectional studies (Selnes, 2015, De Simoni, 2016, Palacios, 2017, Allen, 2006, Grubb, 2000, Orbo, 2018, Ariza, 2006, Di Paola, 2015, Lauer, 2017, Mathias, 2004, Chen, 2016, Sachdev, 2007b, Stamenova, 2018, Himanen, 2005, Schaapsmeerders, 2015b, Palacios, 2013b, Arenth, 2014, Geary, 2010, Palacios, 2011, Baek, 2013, Sugiyama, 2009, Chang, 2010, Jang et al., 2018, Kinnunen, 2011, van der Horn, 2017, Santhanam, 2019) and predictive for (impairments of) function in these cognitive domains in longitudinal studies (Palacios, 2017, Sachdev, 2014, Gu, 2013). Due to the low number of studies included in meta-analyses and high heterogeneity of studies, evidence is weak.

Our results show that smaller brain volumes and larger ventricles have been associated with and were predictive for worse cognitive performance. Especially lower hippocampal volume has repeatedly been associated with worse memory (Selnes, 2015, Allen, 2006, Grubb, 2000, Orbo, 2018, Ariza, 2006, Di Paola, 2015, Lauer, 2017, Mathias, 2004, Chen, 2016, Sachdev, 2007b, Stamenova, 2018, Himanen, 2005, Schaapsmeerders, 2015b, Sachdev, 2014). Since included studies could not compare post-injury with pre-injury results, it is unclear whether volume changes caused by the injury or preexistent atrophy was studied. However, since multiple studies found consonant results regardless of the MRI timing, volumes of the hippocampus and other target regions are probably an important determinant of post-injury memory (impairments).

Diffusion-tensor imaging showed that lower FA in multiple white matter tracts, especially in cingulum and fornix, was associated with worse cognition (De Simoni, 2016, Palacios, 2013b, Geary, 2010, Palacios, 2011, Baek, 2013, Sugiyama, 2009, Chang, 2010, Jang et al., 2018, Kinnunen, 2011, Zuo, 2018, Chiou, 2019, Gu, 2013). FA has mainly been investigated in patients with TBI, so associations after acute onset brain injury from other causes are unclear. Lower FA indicates less intact white matter tracts and low FA values in our study population probably indicate injury induced white matter damage (Le Bihan, 2001). Results of a longitudinal predictive study on diffusivity measures showed that higher anisotropy and lower diffusivity were predictive for worse attention performance, while in cross-sectional studies higher anisotropy and lower diffusivity were always associated with worse performance (Gu, 2013).

Lower FC within the DMN was associated with worse cognitive performance (De Simoni, 2016, Palacios, 2017, Santhanam, 2019, Dall'Acqua, 2017). Of all resting state networks, the DMN was studied most. The DMN comprises the medial prefrontal cortex, posterior cingulate cortex/precuneus, inferior parietal lobe, lateral temporal cortex, and hippocampal formation (Buckner et al., 2008). Although the precise function of the DMN is incompletely understood, it has been established that this network is involved in multiple cognitive functions (Smallwood, 2021). This applies to connectivity within the network (Fox and Raichle, 2007) as well as for the individual brain areas belonging to the network. The posterior cingulate cortex is involved in memory encoding and consolidation (Natu, 2019), while the medial prefrontal cortex participates in error identification (Miller, 2000). Acute onset brain injury may affect nodes of the DMN as well as inter-nodal connections.

Results of three other classes of MRI measures were not straightforward. The association between lesion volume and cognitive performance was inconsistent (Danet, 2015, Exner et al., 2001, Spitz, 2013b, Diao, 2017, Schouten, 2009, Mandzia, 2016). Signs of small vessel disease showed conflicting results. Some studies showed significant correlations (Selnes, 2015, Muir, 2015, Jokinen, 2005, Sachdev, 2014, Vataja, 2003, Pohjasvaara, 2007, Gregoire, 2013, Christ et al., 2019, Divya, 2017), while other did not (Exner et al., 2001, van Rooij, 2017, Auriat, 2019, Yatawara, 2020, Tang, 2011). This type of abnormality is mostly chronic and preexistent (Chojdak-Lukasiewicz, 2021). Arterial spin labeling was only investigated in one study (Ware, 2020).

Our review shows that results from longitudinal predictive studies are not always in accordance with those from cross-sectional studies. This indicates the need for longitudinal studies on potential MRI markers that may predict (impairments of) cognition, since cross-sectional studies are not sufficient.

Our results are consistent with studies in neurodegenerative diseases, where lower fractional anisotropy (Mayo, 2018), lower FC within the DMN (Binnewijzend, 2012), and lower hippocampal volume (Wu, 2019) were associated with poorer cognitive performance, as well.

The broad scope of this review allowed inclusion of a large variability in study populations, studied MRI markers, neuropsychological tests, and timing of MRI and neuropsychological testing, causing substantial heterogeneity across the included studies. Since neuroplasticity and recovery take place on the timescale of weeks or months after acute onset brain injury (Su et al., 2016, Wlodarczyk, 2022), time between onset and MRI or cognitive testing possibly confounded the associations and predictive values described in this review. This review comprised several studies that included the same patients and imaging modalities, which might have led to overestimation of some associations between MRI factors and cognitive outcome measures. However, our meta-analyses never included two studies based on the same data.

A particular strength of this review is the inclusion of studies using neuropsychological tests with known psychometric quality and validity in the acute brain injury population only. We excluded new tests, screening tools, or tests mainly used in research settings. A limitation is that possibilities to perform meta-analyses were limited, because of heterogeneity of the included studies and selective reporting of results. Furthermore, studies eligible for meta-analysis all but two had small sample sizes (n ≤ 40). Although missing data were requested, we may overestimate the pooled correlations in our meta-analyses.

Ultimately, we would want to be able to predict long-term cognitive impairments based on early screening of patients after acute onset brain injury. Based on the results of this systematic review and meta-analysis, some major challenges remain. First, most studies were cross-sectional. Longitudinal studies are needed to be able to predict long-term cognitive outcome based on early screening. Our review shows that longitudinal studies on early MRI markers predictive for long-term cognition were scarce. Grouping and quantifying these results was not possible because no MRI marker was studied longitudinally more than once. International experts recommend early cognitive screening in order to provide cognitive rehabilitation therapy (Cronberg, 2020, Winstein, 2016, Lee, 2019). Second, the additional value of MRI, on top of other potential predictors, such as demographic or neuropsychological measures, has not been addressed. Research has mainly focused on finding group differences and correlations, without taking other possible predictors into account. Third, most studies looked into cognitive functioning instead of impairments. Finally, cut off values of the various proposed MRI markers for predicting impairments in the individual patient are lacking, and external validation of results has not been performed. Future research may focus on finding cut off values of early MRI markers for prediction of long-term cognitive impairments in individual patients and external validation of results.

In conclusion, hippocampal volume, fractional anisotropy in cingulum and fornix, and functional connectivity within the default-mode network are associated with (impairments of) memory, attention, and executive functioning after acute onset brain injury. For clinical implementation, more research is needed on early predictive values of these MRI markers, external validation of results, and cut off values for prediction of long-term cognitive impairments in individual patients.

## Declaration of Competing Interest

The authors declare that they have no known competing financial interests or personal relationships that could have appeared to influence the work reported in this paper.

## Data Availability

Data will be made available on request.
