# Supplementary material for: MRI factors associated with cognitive functioning after acute onset brain injury: Systematic review and meta-analysis
Source: Neuroimage Clin. 2023 Apr 23;38:103415. doi: 10.1016/j.nicl.2023.103415 (PMC10165272; doi:10.1016/j.nicl.2023.103415)
Supplement: Supplementary data 1 [file mmc1.docx]

# MRI factors associated with cognitive functioning after acute onset brain injury: systematic review and meta-analysis

Marlous M.L.H. Verhulst^1,2^; Astrid B. Glimmerveen^1,2^; Caroline M. van Heugten^3,4,5^; Rick C.G. Helmich^6,7^; Jeannette Hofmeijer^1,2^

^1^ Clinical Neurophysiology, University of Twente, Enschede, The Netherlands

^2^ Department of Neurology, Rijnstate Hospital, Arnhem, The Netherlands

^3^ Department of Psychiatry and Neuropsychology, School for Mental Health and Neuroscience, Maastricht University, Maastricht, The Netherlands

^4^ Limburg Brain Injury Center, Maastricht University, Maastricht, The Netherlands

^5^ Department of Neuropsychology and Psychopharmacology, Faculty of Psychology and Neuroscience, Maastricht University, Maastricht, The Netherlands

^6^ Donders Institute for Brain, Cognition, and Behavior, Centre for Cognitive Neuroimaging, Radboud University Nijmegen, Nijmegen, The Netherlands

^7^ Department of Neurology, Centre of Expertise for Parkinson & Movement Disorders, Radboud University Medical Centre, Nijmegen, The Netherlands

**Corresponding author**

Marlous Verhulst ([m.m.l.h.verhulst@utwente.nl](mailto:m.m.l.h.verhulst@utwente.nl))

# Supplementary information (SI)

**Table S1** Scopus search strategy

| **Search item** | **Search terms** |
| --- | --- |
| #1 | (memory[Mesh] OR "spatial memory"[Mesh] OR "memory, episodic"[Mesh] OR "memory, short-term"[Mesh] OR "memory, long-term"[Mesh] OR "memory disorders"[Mesh] OR attention[Mesh] OR "executive function"[Mesh] OR (Cogniti*[tiab] AND (function*[tiab] OR dysfunction*[tiab] OR impairment*[tiab] OR deterioration[tiab] OR disabilit*[tiab] OR recovery[tiab] OR deficit*[tiab] OR disorder*[tiab] OR decline) AND (memor*[tiab] OR recollection[tiab] OR remembrance[tiab] OR recall[tiab] OR recapture[tiab] OR attention[tiab] OR “executive function*”[tiab]))) |
| #2 | ("diffusion magnetic resonance imaging"[Mesh] OR "magnetic resonance imaging"[Mesh] OR "diffusion tensor imaging"[Mesh] OR MRI[tiab] OR “magnetic resonance imaging”[tiab] OR “diffusion-weighted imaging”[tiab] OR “diffusion weighted imaging”[tiab] OR DWI[tiab] OR DTI[tiab] OR “diffusion tensor imaging”[tiab] OR rsfMRI[tiab] OR “resting-state fMRI”[tiab] OR “resting-state functional”[tiab] OR fMRI[tiab]) |
| #3 | (stroke[Mesh] OR “infarction, anterior cerebral artery”[Mesh] OR “infarction, middle cerebral artery”[Mesh] OR “infarction, posterior cerebral artery”[Mesh] OR “heart arrest”[Mesh] OR “out-of-hospital cardiac arrest”[Mesh] OR “post-cardiac-arrest syndrome”[Mesh] OR “hypoxia, brain”[Mesh] OR “hypoxia-ischemia, brain”[Mesh] OR “brain injuries”[Mesh] OR “cerebrovascular trauma”[Mesh] OR “brain injuries, traumatic”[Mesh] OR “brain injuries, diffuse”[Mesh] OR “brain concussion”[Mesh] OR stroke[tiab] OR cva[tiab] OR “brain infarct”[tiab] OR “cerebrovascular accident”[tiab] OR “cardiac arrest”[tiab] OR OHCA[tiab] OR “out-of-hospital cardiac arrest”[tiab] OR “heart arrest”[tiab] OR “postanoxic encephalopathy”[tiab] OR “hypoxic-ischemic”[tiab] OR HIBI[tiab] OR “traumatic brain injury”[tiab] OR TBI[tiab] OR “brain concussion”[tiab] OR “brain injury”[tiab]) |
| #4 | (predict*[tiab] OR diagnos*[tiab] OR associat*[tiab] OR correlat*[tiab] OR relat*[tiab] OR correspond*[tiab] OR link[tiab] OR prognos*[tiab] OR affect*[tiab]) |
| #5 | #1 AND #2 AND #3 AND #4 |
| #6 | ((“adolescent”[Mesh] OR child[Mesh] OR infant[Mesh] OR adolescen*[tiab] OR child*[tiab] OR schoolchild*[tiab] OR infant*[tiab] OR girl*[tiab] OR boy[tiab] OR boys[tiab] OR boyhood[tiab] OR teen[tiab] OR teens[tiab] OR teenager*[tiab] OR youth*[tiab] OR pediatr*[tiab] OR paediatr*[tiab] OR puber*[tiab]) NOT (adult[Mesh] OR adult*[tiab] OR man[tiab] OR men[tiab] OR woman[tiab] OR women[tiab])) |
| #7 | (animals[Mesh] NOT humans[Mesh]) |
| #8 | #5 NOT #6 NOT #7 |
| #9 | #8 AND (2000:2021[pdat]) |
| #10 | #9 NOT (review[Publication type] OR “systematic review”[Publication type] OR “meta-analysis”[Publication type] OR “case reports”[Publication type] OR “randomized controlled trial”[Publication type] OR editorial[Publication type]) |
| #11 | #10 AND (English[Filter]) |

***Table S2*** *PubMed search strategy*

| **Search item** | **Search terms** |
| --- | --- |
| #1 | TITLE-ABS-KEY(Cogniti* AND (memor* OR recollection OR remembrance OR recall OR recapture OR attention OR “executive function”) AND (function* OR dysfunction* OR impairment* OR deterioration OR disability OR recovery OR deficit OR disorder OR decline)) |
| #2 | TITLE-ABS-KEY(MRI OR “magnetic resonance imaging” OR “diffusion-weighted imaging” OR “diffusion weighted imaging” OR DWI OR DTI OR “diffusion tensor imaging” OR rsfMRI OR “resting-state fMRI” OR “resting-state functional” OR fMRI) |
| #3 | TITLE-ABS-KEY(stroke OR cva OR “brain infarct” OR “cerebrovascular accident” OR “cardiac arrest” OR OHCA OR “out-of-hospital cardiac arrest” OR “heart arrest” OR “postanoxic encephalopathy” OR “hypoxic-ischemic” OR HIBI OR “traumatic brain injury” OR TBI OR “brain concussion” OR “acute brain injury”) |
| #4 | TITLE-ABS-KEY(predict* OR diagnos* OR associate* OR correlat* OR relat* OR correspond* OR link* OR prognos* OR affect*) |
| #5 | #1 AND #2 AND #3 AND #4 |
| #6 | #5 AND (EXCLUDE(PUBYEAR,1985) OR EXCLUDE(PUBYEAR,1986) OR EXCLUDE(PUBYEAR,1987) OR EXCLUDE(PUBYEAR,1988) OR EXCLUDE(PUBYEAR,1989) OR EXCLUDE(PUBYEAR,1990) OR EXCLUDE(PUBYEAR,1991) OR EXCLUDE(PUBYEAR,1992) OR EXCLUDE(PUBYEAR,1993) OR EXCLUDE(PUBYEAR,1994) OR EXCLUDE(PUBYEAR,1995) OR EXCLUDE(PUBYEAR,1996) OR EXCLUDE(PUBYEAR,1997) OR EXCLUDE(PUBYEAR,1998) OR EXCLUDE(PUBYEAR,1999)) |
| #7 | #6 AND (EXCLUDE(DOCTYPE,”re”) OR EXCLUDE(DOCTYPE,”ed”) OR EXCLUDE(DOCTYPE,”ch”) OR EXCLUDE(DOCTYPE,”le”) OR EXCLUDE(DOCTYPE,”cr”) OR EXCLUDE(DOCTYPE,”er”)) |
| #8 | #7 AND (LIMIT-TO(LANGUAGE,”English”)) |
| #9 | #8 AND NOT ((KEY(“animal model”) OR KEY(“nonhuman”) OR KEY(“animal experiment”) OR KEY(animals)) AND NOT (KEY(human) OR KEY(humans))) |
| #10 | #9 NOT (review[Publication type] OR “systematic review”[Publication type] OR “meta-analysis”[Publication type] OR “case reports”[Publication type] OR “randomized controlled trial”[Publication type] OR editorial[Publication type]) |
| #11 | #10 AND NOT ((KEY(adolescent) OR KEY(child) OR KEY(“school child”)) AND NOT (KEY(adult) OR KEY(“middle aged”) OR KEY(aged) OR KEY(“aged, 80 and over”) OR KEY(“young adult”) OR KEY(“very elderly”))) |

**Table S3** Characteristics and results of studies on the association between location and severity of damage and (impairments of) memory, attention, or executive functioning

| **Author, year of publication** | **Population**  ***N: population size, A: mean age*** | **Design** | **MRI parameters** | **Timing MRI** | **Cognitive tests** | **Timing cognitive tests** | **Result** |
| --- | --- | --- | --- | --- | --- | --- | --- |
| ***TBI*** |  |  |  |  |  |  |  |
| Datta, 2009 [1] | Mild TBI  *N = 20, A = 35* | Cross-sectional | Lesion volume, lesion intensity, lesion location | 3M – 84M | Memory  CFT, RAVLT  Attention  Digit vigilance test  Executive functioning  WCST, stroop, Tower of London | 3M – 84M | Group differences  Presence of lesions vs. no lesions  *No significant differences on any cognitive test*  Correlation coefficient  Lesion intensity vs. any cognitive test  *No significant correlation* |
| Kurca, 2006 [2] | Mild TBI  *N = 30, A = 32* | Cross-sectional | Lesion volume, number of lesions | < 96H | Memory  CVLT | < 96H | Group differences  Presence of traumatic damage vs. no traumatic damage  *Better performance on CVLT immediate score of interference, CVLT delayed percent retention in no damage group (p<0,05)* |
| Palacios, 2013 [3] | TBI  *N = 26, A = 27* | Cross-sectional | WM/GM contrast | ± 4Y | Memory  RAVLT, digit span, WAIS LNS  Attention/executive functioning  TMT, stroop, SDMT, verbal fluency | ± 4Y | Correlation coefficient  Whole-brain WM/GM intensity contrast vs. RAVLT learning, RAVLT DR  *0,61 ≤ r ≤ 0,62 (p = 0,001)* |
| Spitz, 2013 [4] | TBI  *N = 38, A = 41* | Cross-sectional | Lesion volume, lesion location (FLAIR + SWI) | 2M – 53M | Memory  Doors test, WMS digit span  Attention  SDMT  Executive functioning  COWAT, TMT | 2M – 53M | Group differences  High SWI lesion volume vs. low SWI lesion volume  *Better performance on COWAT in high lesion volume group (p < 0,01)*  High SWI frontal lesion volume vs. low SWI frontal lesion volume  *Better performance on visual memory tests in low lesion group (p < 0,05)*  High FLAIR frontal lesion volume vs. low FLAIR frontal lesion volume  *Better performance on TMT-B in low lesion group (p < 0,05)* |
| ***Stroke*** |  |  |  |  |  |  |  |
| Danet, 2015 [5] | Left ischemic thalamic stroke  *N = 12, A = 53* | Cross-sectional | Damage to MTT, lesion volume | > 3M | Memory  FCSRT, WMS LM, CFT  Executive functioning  WMS digit span, WMS spatial span, TMT, stroop, WAIS symbol search, WAIS lexical fluency | > 3M | Group differences  Damaged MTT vs. intact MTT  *Better performance on FCSRT free and cued recall and recognition, WMS LM DR and recognition in intact MTT group (p < 0,01)*  Correlation coefficient  Total lesion volume vs. FCSRT scores  *r = -0,74 (p < 0,05)* |
| Diao, 2017 [6] | Ischemic stroke in internal capsule  *N = 97, A = 56* | Cross-sectional | Lesion volume, lesion location | > 6M | Memory  RAVLT | > 6M | Correlation coefficient  Lesion location, lesion volume vs. RAVLT  *No significant correlation* |
| Exner, 2001 [7] | Thalamic infarction  *N = 15, A = 56* | Cross-sectional | Lesion volume, lesion location | > 6M | Memory  WMS LM, WMS visual reproduction, WMS paired associates  Attention  TMT, stroop  Executive functioning  WCST, verbal fluency | > 6M | Group differences  Left-sided lesion vs. right-sided lesion  *Better performance on TMT-A in right-sided group (p = 0,029)*  Correlation coefficient  Thalamic lesion volume vs. WMS LM DR, WMS visual reproduction IR  *-0,560 ≤ r ≤ -0,630 (p < 0,05)*  Thalamic lesion volume vs. any attention or executive functioning score  *No significant correlation* |
| Leff, 2009 [8] | Stroke  *N = 210, A = 59* | Cross-sectional | GM density | > 1M | Memory  Digit span | > 1M | Correlation coefficient  GM values in left superior temporal gyrus (posterior) vs. auditory short-term memory  *Significant correlation* |
| Liebermann, 2013 [9] | Thalamic stroke  *N = 19, A = 45* | Cross-sectional | Lesion location | 0 – 120M | Memory  CFT, WMS spatial span, WMS digit span  Executive functioning  Lexical and semantic fluency, stroop, WCST | 0 – 120M | Group differences  Lesions of left centromedian and parafascicular nuclei vs. no lesions in this region  *Better performance on WCST in group without lesions (p < 0,05)*  Lesions of left mediodorsal nuclei vs. no lesions in this region  *Better performance on WCST in group without lesions (p < 0,05)* |
| Liu, 2019 [10] | Subcortical infarct in middle cerebral artery territory  *N = 50, A = 53* | Longitudinal | Lesion location | <7D + 4W + 12W | Memory  RAVLT | <7D + 4W + 12W | Correlation coefficient  Lesion location vs. RAVLT scores at any time point  *No significant correlation* |
| Mandzia, 2016 [11] | TIA and minor ischemic stroke  *N = 92, A = 65* | Longitudinal | Lesion location, lesion volume | < 48H | Memory  CVLT, CFT  Attention  TMT, digit symbol coding  Executive functioning  COWAT, TMT, Clox-1 | 90D | Group differences  Bilateral lesions vs. no or one-sided lesions  *Better performance on executive functioning tests in group with no or one-sided lesions (p = 0,04)* |
| Martinaud, 2009 [12] | Anterior communicating artery aneurysm rupture  *N = 74, A = 47* | Longitudinal | Lesion location (51 ROIs) | 6M – 73M | Memory  Digit span, spatial span, Doors test  Executive functioning  TMT, stroop, verbal fluency, WCST | Baseline | Logistic regression  Lesion of superior part of left superior gyrus vs. initiation deficit  *Correctly classified = 85% (p = 0,02)*  Lesion of left centrum semiovale anterior vs. inhibition deficit  *Correctly classified = 82% (p = 0,02)*  Lesion of left middle gyrus vs. generation deficit  *Correctly classified = 84% (p = 0,02)* |
| Muir, 2015 [13] | Ischemic stroke  *N = 106, A = 64* | Cross-sectional | Stroke involvement in executive network structures, superior longitudinal fasciculus, and lateral cholinergic projections | ± 290D | Executive functioning  TMT | ± 290D | Group differences  0 or 1 errors vs. 2 or more errors on TMT-B set shifting  *More stroke involvement in lateral cholinergic projections in group with 2 or more errors (p = 0,03)*  Linear regressions  Lateral cholinergic projections involvement score vs. TMT difference score, TMT proportion score, TMT quotient score  *0,34 ≤ β ≤ 0,39 (p < 0,0001)*  Left superior longitudinal fasciculus stroke vs. TMT difference score  *β = 0,17 (p = 0,03)*  Right lateral cholinergic projections involvement score vs. TMT difference score, TMT proportion score, TMT quotient score  *0,37 ≤ β ≤ 0,38 (p < 0,0001)*  Left lateral cholinergic projections involvement score vs. TMT difference score  *β = 0,2 (p = 0,007)* |
| Schouten, 2009 [14] | Supratentorial non-lacunar infarct  *N = 86, A = 63* | Longitudinal | Lesion location, lesion volume | 5D – 23D | Memory  RAVLT, Doors test | 1Y | Group differences  Left hemisphere stroke vs. right hemisphere stroke  *Better performance on verbal IR and verbal DR in right hemisphere stroke group (p < 0,01)*  Linear regression  Right-sided lesion vs. verbal IR, verbal DR, verbal recognition  *0,23 ≤ β ≤ 0,28 (p < 0,05)*  Cortical lesion vs. verbal IR, verbal DR, verbal recognition  *0,31 ≤ β ≤ 0,35 (p < 0,01)*  Lesion volume vs. verbal IR, verbal DR, verbal recognition  *-0,25 ≤ β ≤ -0,33 (p < 0,05)* |
| Van Rooij, 2017 [15] | TIA or TNA  *N = 121, A = 65* | Longitudinal | DWI lesions | < 7D | Memory  RAVLT  Executive functioning  Verbal fluency, stroop, Brixton spatial anticipation test | < 7D + 6M | Group differences  Presence of DWI lesions vs. no lesions  *Better performance on executive functioning tests at 7D in group without lesions (p = 0,048)*  *More decline on executive functioning tests between 7D and 6M in group with lesions (p = 0,04)* |

*CFT: Complex Figure Test, Clox-1: clock-drawing task, COWAT: Controlled Oral Word Association Test, CVLT: California Verbal Learning Test, D: days, DR: delayed recall, DWI: diffusion weighted imaging, FCSRT: Free and Cued Selective Reminding Test, FLAIR: fluid attenuated inversion recovery, GM: grey matter, H: hours, IR: immediate recall, LM: logical memory, LNS: letter-number sequencing, M: months, MTT: mammillothalamic tract, RAVLT: Rey Auditory Verbal Learning Test, ROI: region of interest, SDMT: Symbol Digit Modalities Test, SWI: susceptibility weighted imaging, TBI: traumatic brain injury, TIA: transient ischemic attack TMT: Trail Making Test, TNA: transient neurological attack, W: weeks, WAIS: Wechsler Adult Intelligence Scale, WCST: Wisconsin Card Sorting Test, WM: white matter, WMS: Wechsler Memory Scale, Y: years.*

**Table S4** Characteristics and results of studies on the association between cerebral volume/atrophy measures and (impairments of) memory, attention, or executive functioning

| **Author, year of publication** | **Population**  ***N: population size, A: mean age*** | **Design** | **MRI parameters** | **Timing MRI** | **Cognitive tests** | **Timing cognitive tests** | **Result** |
| --- | --- | --- | --- | --- | --- | --- | --- |
| ***PAE*** |  |  |  |  |  |  |  |
| Allen, 2006 [16] | PAE after cardiac arrest  *N = 13, A = 52* | Cross-sectional | Volume residuals (actual value minus expected value) of GM, WM, amygdala, hippocampus | 6M – 10Y | Memory  WMS general memory index, WMS 30-minute DR index, RAVLT, BVRT, CFT | 6M – 10Y | Correlation coefficient  GM volume residual (cerebral, frontal, parietal, temporal) vs. WMS general memory index, WMS DR, RAVLT DR, CFT  *0,583 ≤ r ≤ 844 (p < 0,05)*  WM volume residual (cerebral, frontal, parietal) vs. BVRT errors  *-0,623 ≤ r ≤ -0,718 (p < 0,05)*  Amygdala volume residual vs. WMS DR, RAVLT DR  *0,608 ≤ r ≤ 0,634 (p < 0,05)*  Hippocampus volume residual vs. WMS general memory index, WMS DR, RAVLT DR, CFT  *0,703 ≤ r ≤ 0,875 (p < 0,01)* |
| Di Paola, 2008 [17] | PAE  *N = 5, A = 43* | Cross-sectional | Volume of GM, WM, CSF, hippocampal and parahippocampal cortex | 4M – 33M | Memory  WMS digit span, Corsi block test, Immediate Visual Memory Test, 15-word list, CFT  Executive functioning  MCST, phonological fluency, TMT | 4M – 33M | Correlation coefficient  Hippocampal GM volume vs. any cognitive test  *No significant correlation* |
| Grubb, 2000 [18] | PAE after OHCA  *N = 17, A = 64* | Cross-sectional | Volume of CSF, whole brain, temporal lobe, amygdala and hippocampus | 6M – 22M | Memory  RBMT, DPT | 6M – 22M | Correlation coefficient  Whole brain volume vs. RBMT total, DPT overall  *0,56 ≤ r ≤ 0,67 (p < 0,05)*  Left temporal lobe volume vs. RBMT total, DPT overall  *0,61 ≤ r ≤ 0,71 (p < 0,01)*  Right temporal lobe volume vs. DPT overall  *r = 0,54 (p < 0,05)*  Right amygdala-hippocampus complex volume vs. RBMT total, DPT overall  *0,48 ≤ r ≤ 0,62 (p < 0,05)* |
| Orbo, 2018 [19] | PAE after OHCA  *N = 26, A = 59* | Cross-sectional | Volume residuals of GM, WM, hippocampus, subcortical and cortical ROIs | 3M | Memory  CVLT | 3M | Correlation coefficient  Cortical volume vs. CVLT total learning  *r = 0,64 (p = 0,018)*  Total GM volume vs. CVLT total learning  *r = 0,57 (p = 0,048)*  Left subiculum volume vs. CVLT short DR  *r = 0,58 (p = 0,040)*  Right subiculum volume vs. CVLT short DR  *r = 0,57 (p = 0,042)*  Left presubiculum volume vs. CVLT recognition  *r = 0,63 (p = 0,033)* |
| Stamenova, 2018 [20] | PAE after brief OHCA  *N = 8, A = 55* | Cross-sectional | Volume of hippocampal subfields, cortical and subcortical structures | > 5M | Memory  CANTAB, CFT, CVLT, WMS Verbal Paired Associates, WMS LM  Executive functioning  WAIS digit span, PASAT, TMT, COWAT, CANTAB | > 5M | Correlation coefficient  Right hippocampal volume vs. CVLT IR, CVLT DR  *0,707 ≤ r ≤ 0,826 (p < 0,05)* |
| ***TBI*** |  |  |  |  |  |  |  |
| Ariza, 2006 [21] | TBI  *N = 20, A = 25* | Cross-sectional | Volume of hippocampal head, hippocampal body, hippocampal tail | 6M – 10M | Memory  RAVLT, CFT | 6M – 10M | Correlation coefficient  Left hippocampal head volume vs. RAVLT IR  *r = 0,5 (p = 0,031)* |
| Di Paola, 2015 [22] | Severe TBI  *N = 15, A = 32* | Cross-sectional | Volume of hippocampus | 60D – 887D | Memory  Digit span, 15-word list, prose recall  Executive functioning  MCST, TMT, Tower of London, digit span backward | 60D-887D | Correlation coefficient  Left hippocampal volume vs. 15-word list DR, prose IR, prose DR  *0,425 ≤ r ≤ 0,490 (p < 0,05)* |
| Gale, 2005 [23] | TBI  *N = 9, A = 29* | Cross-sectional | GM concentration | 1Y | Attention  CPT | 1Y | Correlation coefficient  Right frontal, right temporal, right parietal, left cingulate, left frontal GM concentration vs. CPT  *positive r (p < 0,05)* |
| Himanen, 2005 [24] | TBI  *N = 61, A = 29* | Cross-sectional | Volume of hippocampus and lateral ventricles | 27Y – 49Y | Memory  WMS associative learning, BVRT  Attention/executive  TMT, MCST, verbal fluency | 27Y – 49Y | Linear regression  Left hippocampal volume vs. WMS associative learning, MCST errors  *β = 0,35, β = -0,24 (p < 0,05)*  Right hippocampal volume vs. WMS associative learning, MCST perseverative errors  *β = 0,28, β = -0,26 (p < 0,05)*  Lateral ventricle volume vs. BVRT correct/errors, MCST errors, MCST perseverative errors  *0,29 ≤ β ≤ 0,37 (p < 0,01)* |
| Killgore, 2016 [25] | Mild TBI  *N = 26, A = 23* | Cross-sectional | Volume of GM | 2W – 1Y | Executive functioning  D-KEFS | 2W – 1Y | Correlation coefficient  Right fusiform gyrus GM volume vs. D-KEFS design fluency 1 and 2  *0,358 ≤ r ≤ 0,422 (p < 0,05)*  Bilateral ventromedial prefrontal cortex/gyrus rectus GM volume vs. D-KEFS design fluency 1 and 2  *0,417 ≤ r ≤ 0,418 (p < 0,05)* |
| Lauer, 2017 [26] | TBI  *N = 39, A = 35* | Cross-sectional | Volumes of GM, WM and CSF | > 6M | Memory  CANTAB | > 6M | Correlation coefficient  WM volume in right hippocampus vs. CANTAB pattern recognition memory correct  *r = 0,46 (p = 0,004)*  CSF volume in right hippocampus vs. CANTAB pattern recognition memory correct  *r = -0,43 (p = 0,007)*  WM volume in left middle frontal gyrus, right inferior frontal gyrus vs. CANTAB spatial recognition memory latency  *-0,43 ≤ r ≤ -0,47 (p < 0,01)*  GM volume in right inferior temporal gyrus, right precentral gyrus, right fusiform gyrus vs. CANTAB paired associates learning errors  *-0,45 ≤ r ≤ -0,49 (p < 0,01)*  WM volume in right hippocampus, right middle temporal gyrus, right inferior temporal gyrus, left pallidum, right pallidum vs. paired associates learning errors  *-0,43 ≤ r ≤ -0,55 (p < 0,01)*  CSF volume in right inferior temporal gyrus, right inferior frontal gyrus, left superior occipital gyrus vs. CANTAB paired associates learning errors  *0,43 ≤ r ≤ 0,52 (p < 0,01)* |
| Livny, 2017 [27] | TBI  *N = 24, A = 29* | Cross-sectional | Volume of GM and WM | 8M – 80M | Memory  WAIS Arithmetic  Executive functioning  Raven’s progressive matrices, WAIS Similarities | Age 17 + 8-80M | Correlation coefficient  GM volume in left and right cortices vs. Raven’s progressive matrices change score between age 17 and 18-80M after injury  *positive r (p < 0,05)* |
| Mathias, 2004 [28] | Moderate/severe TBI  *N = 25, A = 28* | Cross-sectional | Volume of WM and hippocampus, corpus callosum area | ± 213D | Memory  RAVLT  Attention  TEA  Executive functioning  COWAT, RFFT, WCST | ± 213D | Correlation coefficient  Hippocampal volume vs. RAVLT DR  *r = 0,73 (p < 0,01)* |
| Merkley, 2013 [29] | Severe TBI  *N = 12, A = 23* | Cross-sectional | Volume of cingulate, caudal anterior cingulate, rostral anterior cingulate, total brain and total ventricles, ventricle to brain ratio | 2M – 18M | Memory  ACT | 2M – 18M | Correlation coefficient  Volume of any brain region vs. total ACT score  *No significant correlation* |
| Munivenkatappa, 2019 [30] | Mild TBI  *N = 21, A = 26* | Longitudinal | Volume of cortical and subcortical ROIs | Baseline + 3-4M + 6-7M | Memory  RAVLT, CFT  Attention  DSST  Executive functioning  Animal naming test, spatial span, stroop | Baseline + 3-4M + 6-7M | Correlation coefficient  Right cingulate gyrus volume vs. DSST at baseline + 6-7M  *r =-0,52 (p < 0,05)*  Cingulate gyrus volume vs. CFT IR, RAVLT DR, RAVLT total recall, RAVLT IR at different time points  *0,49 ≤ r ≤ 0,62 (p < 0,05)*  Parietal lobe volume vs. CFT IR at 6-7M  *r = 0,52 (p = 0,039)*  Left parietal lobe volume vs. CFT DR at 3-4M + 6-7M  *0,5 ≤ r ≤ 0,65 (p < 0,05)*  Right temporal lobe volume vs. RAVLT total recall at 3-4M, RAVLT DR at 6-7M  *0,52 ≤ r ≤ 0,65 (p < 0,05)* |
| Ostberg, 2020 [31] | TBI  *N = 114, A = 49* | Longitudinal | Volume and atrophy rate of ROIs | 20D + 238D | Attention  CANTAB | 238D | Linear regression  Precentral gyrus medial segment atrophy rate vs. CANTAB motor screening task  *10% variance explained (p < 0,01)*  Superior frontal gyrus medial segment atrophy rate vs. CANTAB motor screening task  *7% variance explained (p < 0,01)*  Opercular part of inferior frontal gyrus atrophy rate vs. CANTAB motor screening task  *8% variance explained (p < 0,01)*  Supplementary motor cortex atrophy rate vs. CANTAB motor screening task, CANTAB simple reaction time, CANTAB rapid visual information processing  *6 ≤ % variance explained ≤ 16 (p < 0,05)*  Cortical GM atrophy rate vs. CANTAB motor screening task  *8% variance explained (p < 0,01)* |
| Palacios, 2013 [32] | Severe TBI  *N = 26, A = 27* | Cross-sectional | Cortical thickness, volume of hippocampus, brain parenchymal volume | ± 4,2Y | Memory  RAVLT | ± 4,2Y | Correlation coefficient  Cortical thickness in superior frontal cortex and inferior and superior parietal cortex vs. RAVLT DR  *r = 0,70 (p < 0,001)* |
| Spitz, 2013 [33] | TBI  *N = 69, A = 35* | Cross-sectional | Cortical thickness, volume of cortex | 3M – 65M | Memory  Doors test, WMS digit span  Attention  SDMT  Executive functioning  COWAT, TMT | 3M – 65M | Correlation coefficient  Cortical volume in right hemisphere (especially superior temporal and lateral occipital regions) vs. digit span backwards  *r = positive (p < 0,05)*  Cortical volume in right superior parietal region vs. TMT trail B, TMT trail B-A  *r = positive (p < 0,05)* |
| Stewan Feltrin, 2018 [34] | TBI  *N = 24, A = 28* | Longitudinal | Volume of total brain, WM, cortical GM, subcortical GM | 2M + 6M + 12M | Memory  HVLT  Attention/executive functioning  TMT | 2M + 6M + 12M | Correlation coefficient  Total brain volume reduction (% in a year) vs. TMT trail B (6M + 12M)  *0,48 ≤ r ≤ 0,56 (p < 0,05)*  WM volume reduction (% in a year) vs. TMT trail A (12M)  *r = 0,50 (p = 0,03)* |
| Vannorsdall, 2010 [35] | TBI  *N = 14, A = 44* | Cross-sectional | Volume of GM, WM, CSF (corrected for total intracranial volume) | 18M – 366M | Memory  HVLT, BVMT  Executive functioning  TMT, verbal fluency | 18M – 366M | Correlation coefficient  WM/total intracranial volume ratio vs. HVLT word list learning, BVMT visual learning, BVMT recall  *0,56 ≤ r ≤ 0,69 (p<0,05)*  GM/total intracranial volume ratio vs. any cognitive test  *No significant correlation* |
| Ware, 2020 [36] | Moderate to severe TBI  *N = 36, A = 34* | Longitudinal | Jacobian determinant in ROIs (regions with lower CBF than controls) and ventricles | 3M | Memory  RAVLT  Executive functioning  TMT, stroop, digit span, WAIS LNS | 3M + 6M + 12M | Correlation coefficient  Meta-ROI Jacobian vs. any cognitive score at any timepoint  *No significant correlations*  Ventricles Jacobian vs. composite executive functioning score at 3M  *r = -0,48 (p = 0,004)* |
| Wright, 2013 [37] | TBI  *N = 14, A = 29* | Longitudinal | Atrophy of total brain, temporal, frontal, parietal and occipital regions | 8D + 195D | Memory  CFT, selective reminding test  Attention  SDMT  Executive functioning  TMT, COWAT | 12M | Correlation coefficient  Total brain atrophy vs. SDMT (oral)  *r = 0,62 (p < 0,05)*  Right temporal lobe atrophy vs. SDMT (oral), TMT trail B  *r = 0,6, r = -0,65 (p < 0,05)*  Left temporal lobe atrophy vs. SDMT (oral), TMT trail B  *r = 0,49, r = -0,71 (p < 0,05)*  Left frontal lobe atrophy vs. TMT trail B  *r = -0,67 (p < 0,05)* |
| ***Stroke*** |  |  |  |  |  |  |  |
| Auriat, 2019 [38] | Stroke  *N = 30, A = 66* | Cross-sectional | Volume of ventricular CSF and sulcal CSF | 6M – 300M | Memory  CVLT, WMS visual reproduction | 6M – 300M | Correlation coefficient  Ventricular CSF volume vs. memory performance (mean z-score)  *r = -0,527 (p = 0,003)* |
| Chen, 2016 [39] | Thalamic infarction  *N = 37, A = 59* | Cross-sectional | Volume of hippocampal subfields (CA1, CA2/3, CA4/DG, fimbria, fissure, presubiculum and subiculum) | 239±65D | Memory  RAVLT, CFT, WMS digit span backward | 239±65D | Correlation coefficient  Left presubiculum volume vs. CFT complex graphics recall  *r = 0,686 (p = 0,002)*  Left subiculum volume vs. RAVLT short DR  *r = 0,537 (p = 0,022)*  Right presubiculum volume vs. RAVLT short DR, RAVLT long DR, complex graphic recall  *0,638 ≤ r ≤ 0,754 (p < 0,05)* |
| Danet, 2015 [5] | Left thalamic infarction  *N = 12, A = 53* | Cross-sectional | Volume of MMT | 3M – 5Y | Memory  FCSRT, WMS LM, CFT  Executive functioning  WMS digit span, WMS spatial span, TMT, stroop, WAIS symbol search, lexical fluency, WAIS similarities | 3M – 5Y | Correlation coefficient  MMT volume vs. FCSRT 3 free and cued recalls, WMS LM DR  *0,69 ≤ r ≤ 0,80 (p < 0,05)* |
| Diao, 2017 [6] | Ischemic stroke in internal capsule  *N = 97, A = 56* | Cross-sectional | Volume of GM | > 6M | Memory  RAVLT | > 6M | Correlation coefficient  GM volume in right middle frontal gyrus vs. RAVLT total recall  *r = 0,431 (p = 0,003)*  GM volume in left triangular part of inferior frontal gyrus vs. RAVLT total recall  *r = 0,33 (p = 0,029)*  GM volume in right dorsal posterior cingulate cortex vs. RAVLT total recall  *r = 0,387 (p = 0,009)* |
| Jokinen, 2004 [40] | Ischemic stroke  *N = 260, A = 70* | Cross-sectional | MTA | 3M | Memory  WMS LM, WMS visual reproduction, WMS digit span, FOME  Attention/executive functioning  TMT, verbal fluency | 3M | Group differences  No MTA vs. mild MTA vs. moderate/severe MTA  *Better performance in no MTA group (no MTA > mild MTA > moderate/severe MTA) on WMS LM IR, WMS LM DR, WMS visual reproduction IR, WMS visual reproduction DR, FOME total retrieval, TMT-A (p < 0,001)* |
| Jokinen, 2005 [41] | Ischemic stroke  *N = 323, A = 70* | Cross-sectional | Brain atrophy sum score | 3M | Memory  WMS LM, WMS visual reproduction, WMS digit span, FOME  Attention  TMT, stroop  Executive functioning  TMT, stroop, WCST, verbal fluency | 3M | Linear regression  Cortical atrophy vs. TMT-A time, stroop dots time, TMT-B correct, stroop words correct, WCST perseverations, category fluency, WMS LM DR, WMS visual reproduction IR, WMS visual reproduction DR, FOME total recall, FOME DR  *2,2 ≤ % variance explained ≤ 4,7 (p < 0,01)* |
| Liu, 2019 [10] | Subcortical infarct in middle cerebral artery territory  *N = 50, A = 53* | Longitudinal | Atrophy rates of ROIs | <7D + 4W + 12W | Memory  RAVLT | <7D + 4W + 12W | Correlation coefficient  Left thalamic atrophy rate between baseline and 12W vs. change in RAVLT IR between baseline and 12W  *r = 0,450 (p = 0,016)* |
| Munir, 2019 [42] | TIA or minor ischemic stroke  *N = 80, A = 65* | Longitudinal | Volume of total brain, GM and WM, atrophy rate | 48H + 18M + 3Y | Memory  CVLT  Executive functioning  TMT, COWAT | 90D + 1Y + 2Y + 3Y | Linear regression  Atrophy rate vs. memory composite score, executive functioning composite score  *No significant regression coefficients* |
| Sachdev, 2007 [43] | Stroke or TIA  *N = 90, A = 72* | Cross-sectional | Volume of GM, WM, CSF, amygdala, hippocampus, ventricle to brain ratio | 3M – 6M | Memory  WMS LM, WMS visual reproduction, WAIS digit span, WAIS Arithmetic  Attention  WAIS digit span, WMS mental control  Executive functioning  Colour form sorting text, TMT, verbal fluency | 3M – 6M | Correlation coefficient  Right hippocampal volume vs. WMS visual reproduction  *r = 0,169 (p = 0,033)* |
| Sachdev, 2007 [44] | Stroke or TIA  *N = 47, A = 72* | Longitudinal | Volume of amygdala | 3M – 6M | Memory  WMS LM, WMS visual reproduction, WAIS digit span | 3-6M + 1Y | Correlation coefficient  Amygdala volume vs. WMS visual reproduction at 3-6M  *r = 0,352 (p = 0,02)* |
| Sachdev, 2014 [45] | Ischemic stroke or TIA  *N = 183, A = 72* | Longitudinal | Volume of GM, WM, total brain, hippocampus | 3-6M + 3Y | Memory  WMS LM, WMS visual reproduction, WAIS digit span, WAIS Arithmetic  Attention  WAIS digit span, WMS mental control  Executive functioning  Colour form sorting test, TMT, verbal fluency | 3-6M + 1Y + 3Y | Linear regression  Hippocampal volume (3-6M) vs. WMS LM decline (3Y vs. 3-6M)  *β = 0,522, 5,5% variance explained* |
| Schaapsmeerders, 2015 [46] | Ischemic stroke  *N = 176, A = 40* | Cross-sectional | Volume of hippocampus and thalamus | ± 10,1Y | Memory  RAVLT, CFT | ± 10,1Y | Linear regression  Left hippocampal volume vs. CFT IR, CFT DR  *0,9 ≤ β ≤ 1,01 (p < 0,05)* |
| Selnes, 2015 [47] | Cortical and lacunar ischemic infarction  *N = 27, A = 64* | Cross-sectional | Volume of hippocampus | 3M | Memory  WMS LM, RAVLT  Executive functioning  WAIS LNS, stroop | 3M | Correlation coefficient  Hippocampal volume vs. RAVLT IR, WMS LM IR, WMS LM DR  *0,377 ≤ r ≤ 0,578 (p < 0,05)* |
| Vataja, 2003 [48] | Ischemic stroke  *N = 214, A = 70* | Cross-sectional | Brain atrophy | 3M | Executive functioning  WCST, stroop, TMT, verbal fluency | 3M | Group differences  Executive dysfunction vs. no executive dysfunction  *More central and medial temporal lobe atrophy in executive dysfunction group (p < 0,01)*  Odds ratio  Moderate to severe medial temporal lobe atrophy vs. executive dysfunction  *OR = 2,3 (95% CI: 1,0 – 5,2)* |

*ACT: Auditory Consonant Trigrams, BVRT: Benton Visual Retention Test, CANTAB: Cambridge Neuropsychological Test Automated Battery, CFT: Complex Figure Test, COWAT: Controlled Oral Word Association Test, CPT: Cognitive Performance Test, CSF: cerebrospinal fluid, CVLT: California Verbal Learning Test, D: days, D-KEFS: Delis-Kaplan Executive Function System, DPT: Doors and People Test, DR: delayed recall, DSST: Digit Symbol Substitution Test, FCSRT: Free and Cued Selective Reminding Test, FOME: Fuld Object Memory Evaluation, GM: grey matter, H: hours, HVLT: Hopkins Verbal Learning Test, IR: immediate recall, LM: logical memory, LNS: letter-number sequencing, M: months, MCST: Modified Card Sorting Test, MMT: mammillothalamic tract, MTA: medial temporal lobe atrophy, OHCA: out-of-hospital cardiac arrest, OR: odds ratio, PAE: postanoxic encephalopathy, PASAT: Paced Auditory Serial Addition Test, RAVLT: Rey Auditory Verbal Learning Test, RBMT: Rivermead Behavioural Memory Test, RFFT: Ruff Figural Fluency Test, ROI: region of interest, SDMT: Symbol Digit Modalities Test, TBI: traumatic brain injury, TEA: Test of Everyday Attention, TIA: transient ischemic attack, TMT: Trail Making Test, W: weeks, WAIS: Wechsler Adult Intelligence Scale, WCST: Wisconsin Card Sorting Test, WM: white matter, WMS: Wechsler Memory Scale, Y: years.*

**Table S5** Characteristics and results of studies on the association between SVD measures and (impairments of) memory, attention, or executive functioning

| **Author, year of publication** | **Population**  ***N: population size, A: mean age*** | **Design** | **MRI parameters** | **Timing MRI** | **Cognitive tests** | **Timing cognitive tests** | **Result** |
| --- | --- | --- | --- | --- | --- | --- | --- |
| ***Stroke*** |  |  |  |  |  |  |  |
| Auriat, 2019 [38] | Ischemic or hemorrhagic stroke  *N = 30, A = 66* | Cross-sectional | WMH, covert lacunar infarcts | 6M – 300M | Memory  CVLT, WMS visual reproduction | 6M – 300M | Correlation coefficient  Periventricular/deep WMH volume, periventricular/deep covert lacunar infarct volume vs. memory domain score  *No significant correlation* |
| Christ, 2019 [49] | Ischemic stroke or TIA  *N = 66, A = 77* | Longitudinal | CMBs, Fazekas score | Baseline | Memory  Word list learning  Attention  TMT  Executive functioning  Verbal fluency, phonemic fluency, TMT | 6M | Group differences  Any CMBs vs. no CMBs  *Better word list learning, word list recognition, phonemic fluency in no CMBs group (p < 0,01)*  Multilocular CMBs vs. no CMBs  *Better word list learning, word list recall, word list recognition, phonemic fluency in no CMBs group (p < 0,01)* |
| Divya, 2017 [50] | Minor stroke/TIA  *N = 50, A = 65* | Longitudinal | CMBs, Fazekas score | Baseline | Memory  WMS prose recall, WMS draw geometric figures, RAVLT | 3M | Group differences  No periventricular WMHs vs. grade 1 vs. grade 2/3  *No WMH > grade 1 > grade 2/3 performance on RAVLT cumulative learning, WMS visual IR (p < 0,05)*  No deep WMHs vs. grade 1 vs. grade 2/3  *No WMH > grade 1 > grade 2/3 performance on RAVLT cumulative learning, WMS verbal DR (p < 0,05)* |
| Exner, 2001 [7] | Thalamic infarction  *N = 15, A = 56* | Cross-sectional | WMHs | > 6M | Memory  WMS LM, WMS visual reproduction, WMS paired associates  Attention  TMT, stroop, WMS digit span  Executive functioning  WCST, verbal fluency | > 6M | Group differences  WMHs vs. no WMHs  *No significant differences in performance on any cognitive test* |
| Gregoire, 2013 [51] | Ischemic stroke or TIA  *N = 320, A = 64* | Cross-sectional | CMBs, white matter change score |  | Executive functioning  Stroop, verbal fluency, TMT, Color Form Sorting Task, WCST | Within 3M of MRI | Odds ratio  ≥1 strictly lobar CMB vs. executive impairment  *OR = 2,34 (95%CI: 1,08-5,09, p = 0,031)*  ≥5 lobar CMBs vs. executive impairment  *OR = 13,72 (95%CI: 1,55-121,57, p = 0,019)*  Number of strictly lobar CMBs vs. executive impairment  *OR = 1,33 (p < 0,05)*  CMB in parieto-occipital lobes vs. executive impairment  *OR = 3,05 (95%CI: 1,33-6,96, p = 0,008)*  CMB in insula vs. executive impairment  *OR = 5,55 (95%CI: 1,58-19,53, p = 0,008)* |
| Jokinen, 2005 [41] | Ischemic stroke  *N = 323, A = 70* | Cross-sectional | WMHs in target regions (deep, watershed, subcortical, periventricular) | 3M | Memory  WMS digit span, WMS LM, WMS visual reproduction, FOME  Attention  TMT, stroop  Executive functioning  TMT, stroop, WCST, verbal fluency | 3M | Linear regression  WMHs in target regions vs. TMT-A, stroop dots, TMT-B, stroop words, WCST correct, category fluency, WMS visual reproduction IR, WMS visual reproduction DR, FOME DR  *3,5 ≤ % variance explained ≤ 8,2 (p < 0,01)* |
| Muir, 2015 [13] | Ischemic stroke  *N = 106, A = 64* | Cross-sectional | WMHs | Acute/chronic | Executive functioning  TMT | Acute/chronic | Group differences  Errors on TMT-B vs. no errors on TMT-B  *Larger WMH volume as percentage of total intracranial volume in group with errors (p = 0,045)* |
| Pohjasvaara, 2007 [52] | Ischemic stroke  *N = 395, A = 71* | Cross-sectional | WMHs | 3M | Memory  FOME, WMS LM, WMS visual reproduction, WAIS information  Executive functioning  WCST, TMT, stroop, verbal fluency | 3M | Group differences  No or mild WMHs vs. moderate WMHs vs. severe WMHs  *Better performance on IR and DR tests and executive functioning tests in no or mild WMHs group (p < 0,05)* |
| Sachdev, 2014 [45] | Stroke or TIA  *N = 183, A = 72* | Longitudinal | WMHs | 3-6M + 3Y | Memory  WMS LM, WMS visual reproduction, WAIS digit span backward, WAIS arithmetic  Attention  WAIS digit span forward, WMS mental control  Executive functioning  Color Form Sorting Test, TMT, verbal fluency | 3-6M + 1Y + 3Y | Linear regression  WMH volume at 3-6M vs. verbal memory decline between 3-6M and 3Y  *β = -7,28 (p = 0,024)* |
| Selnes, 2015 [47] | Cortical and lacunar ischemic infarction  *N = 27, A = 64* | Cross-sectional | WMHs | 3M | Memory  WMS LM, RAVLT  Executive functioning  Stroop, WAIS LNS | 3M | Correlation coefficient  WMH load vs. stroop 4  *r = -0,42 (p = 0,016)* |
| Tang, 2011 [53] | Ischemic stroke  *N = 127, A = 71* | Longitudinal | CMBs, WMHs | < 7D | Memory  WMS picture recall, WMS visual reproduction  Attention  WMS digit span, WMS visual memory span  Executive functioning  FAB | 3M + 15M | Group differences  Reverters vs. non-reverters from impaired to non-impaired memory  *No significant differences in number/location of CMBs* |
| Van Rooij, 2017 [15] | TIA or TNA  *N = 121, A = 65* | Longitudinal | Lacunes, territorial infarcts, microbleeds, WMHs | < 7D | Memory  RAVLT  Executive functioning  Verbal fluency, stroop, Brixton spatial anticipation test | < 7D + 1M | Correlation coefficient  Silent infarcts, lacunes, microbleeds, WMH severity vs. any cognitive score  *No significant correlation* |
| Vataja, 2003 [48] | Ischemic stroke  *N = 214, A = 70* | Cross-sectional | WMHs | 3M | Executive functioning  WCST, stroop, TMT, verbal fluency | 3M | Group differences  Executive dysfunction vs. no executive dysfunction  *More periventricular WMHs, subcortical WMHs and centrum semiovale WMHs in executive dysfunction group (p < 0,01)*  Executive dysfunction vs. no executive dysfunction  *Higher Fazekas score in executive dysfunction group (p < 0,01)*  Odds ratio  Fazekas deep white matter score vs. executive dysfunction  *OR = 1,6 (95%CI: 1,0-2,6)* |
| Yatawara, 2020 [54] | Ischemic stroke  *N = 346, A = 62* | Longitudinal | WMHs, lacunes, microbleeds | Baseline | Attention  WMS digit span forward  Executive functioning  FAB | 3M – 6M | Odds ratio  Number of microbleeds vs. executive/attention dysfunction  *No significant odds ratio* |

*CI: confidence interval, CMB: cerebral microbleed, CVLT: California Verbal Learning Test, DR: delayed recall, FAB: Frontal Assessment Battery, FOME: Fuld Object Memory Evaluation, IR: immediate recall, LM: logical memory, LNS: letter-number sequencing, M: months, OR: odds ratio, RAVLT: Rey Auditory Verbal Learning Test, TIA: transient ischemic attack, TMT: Trail Making Test, TNA: transient neurological attack, WAIS: Wechsler Adult Intelligence Scale, WCST: Wisconsin Card Sorting Test, WMH: white matter hyperintensity, WMS: Wechsler Memory Scale, Y: years.*

**Table S6** Characteristics and results of studies on the association between DWI measures and (impairments of) memory, attention, or executive functioning

| **Author, year of publication** | **Population**  ***N: population size, A: mean age*** | **Design** | **MRI parameters** | **Timing MRI** | **Cognitive tests** | **Timing cognitive tests** | **Result** |
| --- | --- | --- | --- | --- | --- | --- | --- |
| ***TBI*** |  |  |  |  |  |  |  |
| Arenth, 2014 [55] | Moderate, severe or complicated TBI  *N = 12, A = 32* | Cross-sectional | FA and RD in CC, genu, body and splenium | 1Y – 3Y | Memory  CVLT  Executive functioning  WCST, TMT, COWAT, WAIS similarities, stroop | 1Y – 3Y | Correlation coefficient  FA in CC vs. CVLT total recall, CVLT short-delay recall  *0,596 ≤ r ≤ 0,627 (p < 0,05)*  FA in CC vs. TMT-B, TMT-A  *Negative r (p < 0,05)*  RD in CC vs. TMT-B, TMT-A  *Positive r (p < 0,05)*  FA in splenium vs. CVLT total recall  *r = 0,67 (p = 0,02)*  RD in splenium vs. CVLT false positives  *r = 0,61 (p = 0,04)* |
| Baek, 2013 [56] | TBI  *N = 35, A = 36* | Cross-sectional | Intactness of cingulum, FA, ADC and voxel number of cingulum | 99D – 495D | Memory  MAS | 100D – 502D | Group differences  Bilateral or unilateral intact cingulum vs. bilateral disrupted cingulum  *Higher MAS scores in group with bilateral or unilateral intact cingulum (p < 0,05)*  Correlation coefficient  FA in cingulum vs. MAS score  *r = 0,357 (p = 0,035)*  Voxel number in cingulum vs. MAS score  *r = 0,5 (p = 0,002)* |
| Chang, 2010 [57] | TBI  *N = 9, A = 28* | Cross-sectional | FA and ADC in columns, body, and crus of fornix | 53D – 350D | Memory  MAS | 53D – 350D | Correlation coefficient  FA in fornix body vs. MAS total, MAS short-term, MAS verbal, MAS visual  *0,692 ≤ r ≤ 0,807 (p < 0,05)* |
| Chiou, 2019 [58] | Moderate to severe TBI  *N = 15, A = 46* | Longitudinal | FA change between baseline and FU | ± 86M + 3Y later | Executive functioning  TMT, verbal fluency, stroop | ± 86M + 3Y later | Correlation coefficient  Increased FA in left forceps minor, cingulum, right superior longitudinal fasciculus, forceps major, inferior fronto-occipital fasciculus, uncinate, inferior longitudinal fasciculus vs. improvement on TMT  *Positive r (p < 0,05)* |
| Chung, 2019 [59] | Mild TBI  *N = 19, A = 30* | Cross-sectional | FA, MD, AD, RD, MK, AK, RK, axonal water fraction, intra-axonal diffusivity, extra-axonal AD, extra-axonal RD in FA skeleton and WM ROIs | ± 16D | Memory  WAIS digit span, WAIS LNS | ± 16D | Correlation coefficient  AK in right superior longitudinal fasciculus vs. WAIS digit span backward  *Positive r (p < 0,05)* |
| Dall’Acqua, 2017 [60] | Mild TBI  *N = 49, A = 35* | Longitudinal | FA, SC | Baseline + 1Y | Memory  WAIS digit span, RAVLT  Attention  TAP | Baseline + 1Y | Correlation coefficient  SC recovery in 19-edge subnetwork (frontal part of left hemisphere) between baseline and 1Y vs. RAVLT recall score  *r = 0,348 (p = 0,009)*  SC recovery in 18-edge subnetwork (right hemisphere) between baseline and 1Y vs. RT in TAP auditory divided attention test  *r = -0,262 (p = 0,039)* |
| De Simoni, 2016 [61] | TBI  *N = 19, A = 39* | Longitudinal | FA, MD, AD, RD in cingulum | Baseline + FU | Memory  CANTAB  Attention  CANTAB | Baseline + FU | Correlation coefficient  FA in right parahippocampal subdivision of cingulum vs. CANTAB paired associated learning at baseline  *r = -0,68 (p = 0,015)* |
| De Simoni, 2018 [62] | Moderate to severe TBI  *N = 42, A = 41* | Cross-sectional | FA and MD in tracts between caudate and anterior cingulate cortex | > 6M | Memory  WMS LM, People test  Executive functioning  TMT, stroop | > 6M | Correlation coefficient  FA in tracts from right anterior caudate, right/left posterior caudate to anterior cingulate cortex vs. stroop  *-0,323 ≤ r ≤ -0,495 (p < 0,05)*  FA in tracts from left anterior caudate to anterior cingulate cortex vs. stroop  *No significant correlations*  MD in tracts from right/left posterior caudate to anterior cingulate cortex vs. stroop  *0,230 ≤ r ≤ 0,451 (p < 0,1)*  MD in tracts from right/left anterior caudate to anterior cingulate cortex vs. stroop  *No significant correlations* |
| Fagerholm, 2015 [63] | Moderate to severe TBI  *N = 52, A = 37* | Cross-sectional | Graph metrics | ± 38M | Memory  People test  Executive functioning  TMT | ± 38M | Linear regression  Eigenvector centrality in left peri-callosal region, right superior frontal gyrus, left thalamus, left caudate, left insula, right cingulate cortex vs. Executive functioning  *Highest β (2SD higher than next best), 2% variance explained*  Betweenness centrality in left superior frontal gyrus, left orbitofrontal cortex, left caudate vs. associative memory  *Highest β, <1% variance explained* |
| Geary, 2010 [64] | Mild closed head TBI  *N = 40, A = 35* | Cross-sectional | FA in WM ROIs | > 6M | Memory  CVLT | > 6M | Linear regression  FA in left uncinate fasciculus, left superior longitudinal fasciculus vs. CVLT trial 1  *9-14% variance explained (p < 0,05)*  Correlation coefficient  FA in left anterior corona radiata vs. CVLT trial 2, trial 3, trial 4, list B  *0,319 ≤ r ≤ 0,376 (p < 0,05)*  FA in right anterior corona radiata vs. CVLT trial 2, list B  *0,346 ≤ r ≤ 0,353 (p < 0,05)*  FA in left forceps minor vs. CVLT trial 1, trial 2, trial 4, list B  *0,321 ≤ r ≤ 0,452 (p < 0,05)*  FA in right forceps major vs. CVLT trial 2  *r = 0,327 (p < 0,05)*  FA in left uncinate fasciculus vs. CVLT trial 1, trial 2, trial 4, list B  *r = 0,336 (p < 0,05)*  FA in right cingulum vs. CVLT trial 1  *r = 0,316 (p < 0,05)*  FA in genu corpus callosum vs. CVLT list B  *r = 0,386 (p < 0,05)* |
| Grossman, 2013 [65] | Mild TBI  *N = 20, A = 35* | Longitudinal | MK, FA, MD in thalamus, putamen, caudate, external capsule, optic radiations, corpus callosum, cingulum, centrum semiovale | Baseline + 9M | Memory  CVLT, CFT, WMS LNS  Attention  WAIS digit span, TMT  Executive functioning  Stroop | Baseline + 9M | Correlation coefficient  MK in thalamus vs. digit span at baseline  *r = 0,62 (p = 0,01)*  MD in external capsule, corpus callosum, total WM vs. CVLT total recall at baseline  *0,54 ≤ r ≤ 0,57 (p < 0,05)*  MK in cingulum, centrum semiovale, total WM vs. CFT short term recall at baseline  *-0,54 ≤ r ≤ -0,60 (p < 0,05)*  Any DTI measure vs. any cognitive test score at 9M  *No significant correlations* |
| Gu, 2013 [66] | Closed head TBI  *N = 15, A = 35* | Longitudinal | FA, MD, AD, and RD in posterior limb of internal capsule, uncinate fasciculus, anterior corona radiate, superior longitudinal fasciculus, inferior longitudinal fasciculus, genu of CC, body of CC, splenium of CC, cingulum bundle | < 7D | Memory  CVLT, WAIS digit span, WAIS digit symbol test  Attention  Stroop | 12M – 26M | Correlation coefficient  AD in uncinate fasciculus vs. working memory score  *r = 0,651 (p = 0,009)*  FA in cingulum bundle, uncinate fasciculus, superior longitudinal fasciculus, inferior longitudinal fasciculus vs. working memory score  *0,527 ≤ r ≤ 0,700 (p < 0,05)*  RD in cingulum bundle, superior longitudinal fasciculus, inferior longitudinal fasciculus vs. working memory score  *-0,603 ≤ r ≤ -0,754 (p < 0,05)*  MD in superior longitudinal fasciculus, inferior longitudinal fasciculus vs. Working memory score  *-0,630 ≤ r ≤ -0,681 (p < 0,05)*  RD in anterior corona radiate, superior longitudinal fasciculus, inferior longitudinal fasciculus vs. attention score  *0,570 ≤ r ≤ 0,655 (p < 0,05)*  MD in anterior corona radiate, superior longitudinal fasciculus, inferior longitudinal fasciculus vs. attention score  *0,575 ≤ r ≤ 0,675 (p < 0,05)*  FA in posterior limb of internal capsule, anterior corona radiate, superior longitudinal fasciculus, inferior longitudinal fasciculus vs. attention score  *-0,516 ≤ r ≤ -0,569 (p < 0,05)* |
| Hellyer, 2013 [67] | TBI  *N = 65, A = 35* | Cross-sectional | FA, MO, MD, and RD | > 2M | Memory  People test  Executive functioning  TMT | > 2M | Correlation coefficient  Mean MO vs. executive functioning score  *r = 0,53 (p < 0,01)*  MD in left cingulum, corpus callosum body, left anterior thalamic radiation vs. executive functioning score  *0,54 ≤ r ≤ 0,57 (p < 0,01)* |
| Jang, 2018 [68] | TBI  *N = 86, A = 45* | Cross-sectional | FA and fiber volume of fornix, discontinuation of fornix | ± 6M | Memory  MAS | ± 6M | Group differences  Intactness of fornix vs. global memory  *Better performance in group with both sides intact fornix vs. one side intact and no side intact (p = 0,009, p=0,005)*  Correlation coefficient  FA in fornix vs. global memory  *r = 0,303 (p = 0,006)*  Fiber volume of fornix vs. global memory  *r = 0,271 (p = 0,014)* |
| Kinnunen, 2011 [69] | TBI  *N = 28, A = 39* | Cross-sectional | FA, MD, AD, and RD in WM skeleton | ± 25M | Memory  People test  Executive functioning  TMT, D-KEFS, letter fluency | ± 25M | Correlation coefficient  FA in fornix vs. associative learning and memory  *r = 0,48 (p < 0,001)*  MD in left superior frontal WM vs. TMT set-shifting  *r = 0,75 (p < 0,001)*  RD in right posterior/medial parietal WM vs. TMT set-shifting  *r = 0,53 (p < 0,01)* |
| Kondo, 2010 [70] | Severe TBI  *N = 14, A = 24* | Cross-sectional | FA maps | > 3M | Memory  RBMT | > 3M | Correlation coefficient  FA in left parahippocampal gyrus, left anterior cingulate, left inferior parietal lobe vs. prospective memory failure score  *0,89 ≤ r ≤ 0,93 (p < 0,001)* |
| Kraus, 2007 [71] | TBI  *N = 37, A = 35* | Cross-sectional | FA, RD, and AD in ROIs, WM load | > 6M | Memory  CVLT, BVMT  Attention  WMS digit span, WMS spatial span, TMT, CPT  Executive functioning  Tower of London, stroop, PASAT, TMT, CPT, COWAT, RFFT, WMS digit span, WMS spatial span | > 6M | Correlation coefficient  White matter load vs. executive functioning domain score  *r = -0,41 (p = 0,002)*  White matter load vs. memory domain score  *r = -0,40 (p < 0,001)*  White matter load vs. attention domain score  *No significant correlation*  FA in body of CC, splenium of CC, corticospinal tracts, external capsule, inferior fronto-occipital fasciculus, anterior corona radiate, posterior corona radiate, superior longitudinal fasciculus, sagittal striatum, cingulum fibres vs. executive functioning domain score  *Negative r (p < 0,05)*  FA in forceps major, forceps minor vs. executive functioning domain score  *Positive r (p < 0,05)*  FA in body of CC, splenium of CC, forceps major, forceps minor, inferior fronto-occipital fasciculus, posterior corona radiata, sagittal striatum, cingulum fibres vs. memory domain score  *Negative r (p < 0,05)*  FA in forceps major vs. attention domain score  *Negative r (p = 0,022)*  FA in posterior corona radiata vs. attention domain score  *Positive r (p = 0,046)* |
| Kuceyeski, 2011 [72] | Mild TBI  *N = 15, A = 35* | Cross-sectional | Importance weighted severity score for characteristic path length, efficiency, and spectral radius, tract probability count score, white matter probability score | ± 20M | Memory  CVLT | ± 20M | Correlation coefficient  Any weighted or probability score vs. CVLT DR, total recall, recognition  *No significant correlation* |
| Munivenkatappa, 2016 [73] | Mild TBI  *N = 21, A = 26* | Longitudinal | FA, MD, RD, and AD in thalamus | Baseline + 3-4M + 6-7M | Memory  RAVLT, CFT  Attention  DSST  Executive functioning  Animal naming test, spatial span, stroop | Baseline + 3-4M + 6-7M | Correlation coefficient  MD, RD, AD in thalamus vs. CFT DR at baseline  *0,539 ≤ r ≤ 566 (p < 0,05)*  FA in thalamus vs. DSST at 6-7M  *r = 0,609 (p = 0,008)*  MD, RD, AD in thalamus vs. DSST at 6-7M  *-0,553 ≤ r ≤ -0,656 (p < 0,05)* |
| Munivenkatappa, 2017 [74] | Mild TBI  *N = 21, A = 26* | Longitudinal | FA, MD, RD, AD | Baseline + 3-4M + 6-8M | Memory  RAVLT, CFT  Attention  DSST  Executive functioning  Animal naming test, spatial span, stroop | Baseline + 3-4M + 6-8M | Correlation coefficient  MD, RD, AD in frontal lobe vs. spatial span at baseline  *-0,540 ≤ r ≤ -0,549 (p < 0,05)*  MD, RD in thalamus vs. CFT DR at baseline  *0,539 ≤ r ≤ 0,551 (p < 0,05)*  MD, RD, AD in temporal lobe vs. RAVLT at 3-4M  *-0,495 ≤ r ≤ -0,532 (p < 0,05)*  MD, RD, AD in thalamus vs. DSST at 6-8M  *-0,553 ≤ r ≤ -0,578 (p < 0,05)*  MD, RD, AD in thalamus vs. RAVLT at 6-8M  *0,532 ≤ r ≤ 0,551 (p < 0,05)*  FA in thalamus vs. DSST at 6-8M  *r = 0,619 (p = 0,008)* |
| Owens, 2017 [75] | Moderate to very severe TBI  *N = 15, A = 39* | Cross-sectional | FA and MD in superolateral branches of medial forebrain bundle | 0M – 88M | Memory  WAIS digit span  Attention  SDMT, TMT, digit span | 0M – 88M | Correlation coefficient  FA, MD in superolateral branches of medial forebrain bundle vs. any cognitive measure  *No significant correlation* |
| Palacios, 2011 [76] | Severe TBI  *N = 15, A = 24* | Cross-sectional | FA in skeleton | 86D – 660D | Memory  WAIS digit span, WAIS LNS, RBMT | 86D – 660D | Correlation coefficient  FA in skeletonized corpus callosum, skeletonized fornix vs. RBMT  *0,54 ≤ r ≤ 0,71 (p < 0,05)* |
| Palacios, 2013 [32] | Severe TBI  *N = 26, A = 27* | Cross-sectional | FA in skeleton, global FA | ± 4Y | Memory  RAVLT | ± 4Y | Correlation coefficient  FA in left hippocampus, cingulum, parietal part of superior longitudinal fasciculi vs. RAVLT DR  *Mean r = 0,59 (p < 0,001)* |
| Palacios, 2013 [3] | TBI  *N = 26, A = 27* | Cross-sectional | FA in skeleton | ± 4Y | Memory  RAVLT, digit span, WAIS LNS  Attention  TMT, stroop, SDMT, fluency (semantic, phonemic) | ± 4Y | Correlation coefficient  Mean FA in skeleton vs. RAVLT learning, RAVLT DR  *0,53 ≤ r ≤ 0,59 (p< 0,01)* |
| Rabinowitz, 2018 [77] | Moderate to severe TBI  *N = 46, A = 35* | Longitudinal | FA maps, DAI score | Baseline | Memory  RAVLT  Executive functioning  WAIS LNS, WAIS digit span, COWAT, TMT, stroop | 3M + 6M + 12M | Mixed effects model  DAI score (fixed) vs. executive functioning composite score  *5,9% variance explained (p < 0,005)* |
| Rajagopalan, 2019 [78] | Moderate to severe TBI  *N = 17, A = 28* | Cross-sectional | FA, MD, AD, RD in whole brain and left and right WM | ± 25M | Memory  HVLT  Executive functioning  TMT | ± 25M | Linear regression  FA in WM skeleton vs. executive functioning composite score  *19,5% variance explained (p = 0,02)* |
| Shah, 2012 [79] | Severe TBI  *N = 13, A = 22* | Cross-sectional | FA and ADC in ventral striatum | 6M | Executive functioning  COWAT, TMT | 6M | Correlation coefficient  FA in right ventral striatum vs. COWAT  *r = 0,74 (p = 0,006)*  FA in right ventral striatum vs. time to complete TMT-B  *r = -0,70 (p = 0,011)* |
| Solmaz, 2017 [80] | Moderate to severe TBI  *N = 40, A = -* | Cross-sectional | DISC | 3M | Memory  RAVLT  Executive functioning  COWAT, TMT, stroop, WMS digit span, WMS LNS | 3M | Correlation coefficient  DISC based on edge betweenness centrality, change in efficiency vs. executive functioning composite score  *-0,40 ≤ r ≤ -0,41 (p < 0,05)*  DISC based on edge betweenness centrality, uniform weights, risk ratio vs. verbal learning  *-0,37 ≤ r ≤ -0,42 (p < 0,05)* |
| Spitz, 2013 [81] | TBI  *N = 36, A = 39* | Cross-sectional | FA in WM tracts | 2M – 53M | Memory  Doors test, WMS digit span  Attention  SDMT  Executive functioning  COWAT, TMT | 2M – 53M | Correlation coefficient  FA in left superior longitudinal fasciculus vs. COWAT  *r = 0,35 (p < 0,05)*  FA in right internal capsule vs. TMT trail B  *r = -0,41 (p < 0,05)*  FA in CC, right superior longitudinal fasciculus, right interior longitudinal fasciculus vs. SDMT  *0,35 ≤ r ≤ 0,37 (p < 0,05)* |
| Sugiyama, 2009 [82] | TBI  *N = 11, A = 35* | Cross-sectional | FA maps | > 10M | Memory  WMS, RBMT  Attention  TMT  Executive functioning  WCST | > 10M | Correlation coefficient  FA in right frontal lobe WM, right caudate nucleus, left temporal pole, left insula vs. TMT-A  *Positive r (p < 0,05)*  FA in left supramarginal gyrus, right temporal lobe WM, right middle frontal gyrus, right orbital gyrus, right superior frontal gyrus, left occipital gyrus, right hippocampus, left hippocampus, right middle frontal gyrus, left temporal lobe WM, right cingulum, right postcentral gyrus vs. TMT-B  *Positive r (p < 0,05)*  FA in right cingulum, left cingulum vs. verbal memory  *Positive r (p < 0,01)*  FA in left amygdala, right inferior parietal lobule, right cerebral peduncle vs. DR  *Positive r (p < 0,05)*  FA in right precentral gyrus vs. WCST perseverative errors of Nelson  *Positive r (p = 0,001)* |
| Van der Horn, 2017 [83] | Mild TBI  *N = 53, A = 33* | Longitudinal | Local and global graph measures | 4W | Memory  WAIS digit span, RAVLT | 3M | Correlation coefficient  Betweenness centrality of opercular parts of left inferior frontal gyrus vs. RAVLT IR  *r = -0,57 (p = 0,006)*  Betweenness centrality of left superior temporal gyrus vs. RAVLT DR  *r = -0,49 (p = 0,03)* |
| Wallace, 2020 [84] | TBI  *N = 31, A = 44* | Cross-sectional | FA and MD in genu, corpus callosum, fornix and superior longitudinal fasciculus | ± 200D | Memory  WMS LM, WMS visual reproduction  Executive functioning  COWAT | ± 200D | Correlation coefficient  FA and MD in any ROI vs. any cognitive test score  *No significant correlation* |
| Yamagata, 2020 [85] | TBI  *N = 18, A = 42* | Cross-sectional | FA, AD, and RD of skeleton | 333D – 2395D | Attention/executive functioning  FAB, verbal fluency | 333D – 2395D | Correlation coefficient  FA values in widespread WM regions vs. phonemic verbal fluency  *Positive r (p < 0,05)*  FA values in widespread WM regions vs. semantic verbal fluency  *No significant correlations* |
| Yoo, 2014 [86] | TBI  *N = 20, A = 34* | Cross-sectional | Connection between injured cingulum and brainstem cholinergic nuclei | > 3M | Memory  MAS | > 3M | Group differences  No neural connection vs. presence of neural connection  *Better short term memory performance in neural connection group (p = 0,02)* |
| ***Stroke*** |  |  |  |  |  |  |  |
| Schaapsmeerders, 2015 [87] | Ischemic stroke  *N = 146, A = 40* | Cross-sectional | FA and MD in left and right hippocampus and thalamus | ± 10Y | Memory  RAVLT | ± 10Y | Group differences  Low vs. high MD in hippocampus and thalamus  *No significant differences in memory performance* |
| Zuo, 2018 [88] | Mild stroke with basal ganglia infarcts  *N = 33, A = 51* | Cross-sectional | FA in WM ROIs | 10D – 14D | Memory  RAVLT, CFT  Attention/executive functioning  TMT, stroop | < 10D | Correlation coefficient  FA in right external capsule, right fornix vs. CFT DR  *0,357 ≤ r ≤ 0,469 (p < 0,05)*  Linear regression  FA in right fornix vs. CFT DR  *β = 0,002 (p < 0,05)* |

*AD: axial diffusivity, ADC: apparent diffusion coefficient, AK: axial kurtosis, BVMT: Brief Visuospatial Memory Test, CANTAB: Cambridge Neuropsychological Test Automated Battery, CC: corpus callosum, CFT: Complex Figure Test, COWAT: Controlled Oral Word Association Test, CPT: Continuous Performance Test, CVLT: California Verbal Learning Test, D: days, DAI: diffuse axonal injury, DISC: disruption index of the structural connectome, D-KEFS: Delis-Kaplan Executive Function System, DSST: Digit Symbol Substitution Test, FA: fractional anisotropy, FAB: Frontal Assessment Battery, FU: follow-up, HVLT: Hopkins Verbal Learning Test, LM: logical memory, LNS: letter-number sequencing, M: months, MAS: Memory Assessment Scale, MD: mean diffusivity, MK: mean kurtosis, MO: mode anisotropy, PASAT: Paced Auditory Serial Addition Test, RAVLT: Rey Auditory Verbal Learning Test, RBMT: Rivermead Behavioural Memory Test, RD: radial diffusivity, RFFT: Ruff Figural Fluency Test, RK: radial kurtosis, ROI: region of interest, SC: structural connectivity, SDMT: Symbol Digit Modalities Test, TAP: Test of Attentional Performance, TBI: traumatic brain injury, TMT: Trail Making Test, W: weeks, WAIS: Wechsler Adult Intelligence Scale, WCST: Wisconsin Card Sorting Test, WM: white matter, WMS: Wechsler Memory Scale, Y: years.*

**Table S7** Characteristics and results of studies on the association between rsfMRI measures and (impairments of) memory, attention, or executive functioning

| **Author, year of publication** | **Population**  ***N: population size, A: mean age*** | **Design** | **MRI parameters** | **Timing MRI** | **Cognitive tests** | **Timing cognitive tests** | **Result** |
| --- | --- | --- | --- | --- | --- | --- | --- |
| ***TBI*** |  |  |  |  |  |  |  |
| Bernier, 2017 [89] | Moderate to severe TBI  *N = 19, A = 30* | Cross-sectional | FC within and between DMN and task-related network | > 1Y | Memory  WAIS digit span  Executive functioning  TMT | > 1Y | Correlation coefficient  FC within or between networks during rest vs. any cognitive test score  *No significant correlations* |
| Dall’Acqua, 2017 [60] | Mild TBI  *N = 49, A = 35* | Longitudinal | FC across 90 ROIs | Baseline + 1Y | Memory  WAIS digit span, RAVLT  Attention  TAP | Baseline + 1Y | Correlation coefficient  FC recovery in 15-edge subnetwork (including edges from DMN) between baseline and 1Y vs. RT in TAP visual divided attention test  *r = 0,333 (p = 0,012)*  FC recovery in 15-edge subnetwork (including edges from DMN) between baseline and 1Y vs. working memory score  *r = -0,35 (p = 0,008)* |
| De Simoni, 2016 [61] | TBI  *N = 19, A = 39* | Longitudinal | FC between posterior cingulate cortex and ROIs | Baseline + FU | Memory  CANTAB  Attention  CANTAB | Baseline + FU | Correlation coefficient  FC between posterior cingulate cortex and parahippocampus vs. CANTAB paired associates learning task, RT on CANTAB pattern recognition memory performance at baseline  *-0,57 ≤ r ≤ -0,72 (p < 0,05)* |
| De Simoni, 2018 [62] | Moderate to severe TBI  *N = 42, A = 41* | Cross-sectional | FC in 12 striatal subdivisions | > 6M | Memory  WMS LM, People test  Executive functioning  TMT, stroop | > 6M | Correlation coefficient  FC in right anterior caudate vs. stroop inhibition-switching  *r = -0,592 (p = 0,001)*  FC in right anterior caudate vs. Peoples test DR  *r = 0,421 (p = 0,047)*  FC in right posterior caudate vs. stroop inhibition, stroop inhibition-switching  *-0,492 ≤ r ≤ -0,508 (p < 0,05)* |
| Palacios, 2017 [90] | Mild TBI  *N = 75, A = 32* | Longitudinal | FC in and between RSNs | 5D – 18D | Memory  CVLT  Attention/executive functioning  TMT | 6M | Correlation coefficient  FC in DMN, FC in SN, FC in DAN vs. TMT-A  *Positive r (p < 0,05)*  FC in OFN vs. TMT B-A  *Positive r (p < 0,05)*  FC in OCN vs. learning memory  *Positive r (p < 0,05)*  FC between BGN and OFN vs. TMT B-A  *Negative r (p < 0,05)* |
| Santhanam, 2019 [91] | Mild TBI  *N = 51, A = 32* | Cross-sectional | FC in DMN | ± 23M | Memory  BVMT | ± 23M | Correlation coefficient  FC in posterior cingulate cortex vs. BVMT total recall, BVMT DR  *0,345 ≤ r ≤ 0,352 (p < 0,05)* |
| Shumskaya, 2017 [92] | TBI  *N = 43, A = 42* | Cross-sectional | FC in RSNs | > 1Y | Memory  RAVLT, RBMT, location learning test  Attention  Paced serial addition test, TAP  Executive functioning  Brixton spatial anticipation test, TMT, stroop, WAIS LNS | > 1Y | Correlation coefficient  FC in sensorimotor network vs. attention composite score  *Positive r (p < 0,05)*  Linear regression  FC in sensorimotor network vs. attention composite score  *p < 0,001* |
| Xiong, 2016 [93] | Mild TBI  *N = 32, A = 33* | Cross-sectional | Amplitudes of low frequency fluctuations, FC between 45 ROIs | Chronic phase | Memory  WAIS working memory index | Chronic phase | Correlation coefficient  Amplitudes of low frequency fluctuations in cingulate gyrus vs. WAIS working memory index  *r = 0,423 (p < 0,05)*  FC between left thalamus and left middle frontal gyrus vs. WAIS WMI  *r = 0,381 (p < 0,05)* |
| Xu, 2018 [94] | Mild TBI  *N = 50, A = 37* | Longitudinal | FC between caudate regions | <7D + 1M | Memory  WAIS digit span, digit symbol coding | <7D + 1M | Correlation coefficient  FC between caudate regions vs. memory scores  *No significant correlation* |
| ***Stroke*** |  |  |  |  |  |  |  |
| Liu, 2017 [95] | Right hemisphere stroke  *N = 27, A = 57* | Cross-sectional | FC between DMN and DAN, and other ROIs | < 12M | Memory  WMS digit span, WMS addition, WMS picture, WMS recognition, WMS visual reproduction, WMS associative, WMS learning, WMS touch, WMS comprehension, WMS recital | < 12M | Correlation coefficient  FC between DMN and right medial frontal gyrus vs. WMS total score  *positive r (p < 0,001)*  FC between DMN and right cingulate gyrus, left claustrum, left inferior frontal gyrus vs. WMS total score  *negative r (p < 0,001)*  FC between DAN and right precentral gyrus vs. WMS total score  *positive r (p < 0,001)* |
| Peng, 2016 [96] | Subcortical stroke  *N = 32, A = 60* | Cross-sectional | Regional Homogeneity maps | > 3M | Memory  RAVLT, CFT  Attention  WAIS digit span, SDMT  Executive functioning  WAIS digit span, TMT | > 3M | Correlation coefficient  Regional Homogeneity of bilateral anterior cingulate cortex vs. CFT DR, SDMT  *0,397 ≤ r ≤ 0,399 (p < 0,05)*  Regional Homogeneity of left posterior cingulate cortex/precuneus vs. Digit span forward  *R = 0,485 (p = 0,009)* |
| Tuladhar, 2013 [97] | Ischemic stroke  *N = 20, A = 55* | Cross-sectional | FC in and between ROIs | 9W – 12W | Memory  CVLT | 6W – 8W | Correlation coefficient  FC between ROIs vs. any cognitive test score  *No significant correlation* |
| Yao, 2020 [98] | Stroke with basal ganglia damage  *N = 14, A = 61* | Cross-sectional | Voxel-mirrored homotopic connectivity, degree centrality index | 1W – 3M | Memory  WMS | 1W – 3M | Correlation coefficient  Voxel-mirrored homotopic connectivity in middle temporal gyrus vs. WMS memory quotient  *r = 0,524 (p = 0,001)*  Degree centrality in right supramarginal gyrus vs. WMS memory quotient  *r = 0,428 (p = 0,01)* |

*BGN: basal ganglia network, BVMT: Brief Visuospatial Memory Test, CANTAB: Cambridge Neuropsychological Test Automated Battery, CVLT: California Verbal Learning Test, D: days, DAN: dorsal attention network, DMN: default-mode network, DR: delayed recall, FC: functional connectivity, FCSRT: Free and Cued Selective Reminding Test, FU: follow-up, IR: immediate recall, LM: logical memory, LNS: letter-number sequencing, M: months, OCN: occipital-cerebellar network, OFN: orbitofrontal network, RAVLT: Rey Auditory Verbal Learning Test, RBMT: Rivermead Behavioural Memory Test, ROI: region of interest, RSN: resting-state network, RT: reaction time, SDMT: Symbol Digit Modalities Test, SN: salience network, TAP: Test of Attentional Performance, TBI: traumatic brain injury, TMT: Trail Making Test, W: weeks, WAIS: Wechsler Adult Intelligence Scale, WMS: Wechsler Memory Scale, Y: years.*

**Table S8** Characteristics and results of studies on the association between ASL measures and (impairments of) memory, attention, or executive functioning

| **Author, year of publication** | **Population**  ***N: population size, A: mean age*** | **Design** | **MRI parameters** | **Timing MRI** | **Cognitive tests** | **Timing cognitive tests** | **Result** |
| --- | --- | --- | --- | --- | --- | --- | --- |
| ***TBI*** |  |  |  |  |  |  |  |
| Ware, 2020 [36] | Moderate to severe TBI  *N = 36, A = 34* | Longitudinal | CBF in ROIs (regions with lower CBF than controls) | 3M | Memory  RAVLT  Executive functioning  TMT, stroop, digit span, WAIS LNS | 3M + 6M + 12M | Correlation coefficient  Meta-ROI CBF vs. composite executive functioning score at 3M  *r = 0,43 (p = 0,01)*  Meta-ROI CBF vs. cognitive scores at 6-12M  *No significant correlations* |

*CBF: cerebral blood flow, LNS: letter-number sequencing, M: months, RAVLT: Rey Auditory Verbal Learning Test, ROI: region of interest, TBI: traumatic brain injury, TMT: Trail Making Test, WAIS: Wechsler Adult Intelligence Scale.*


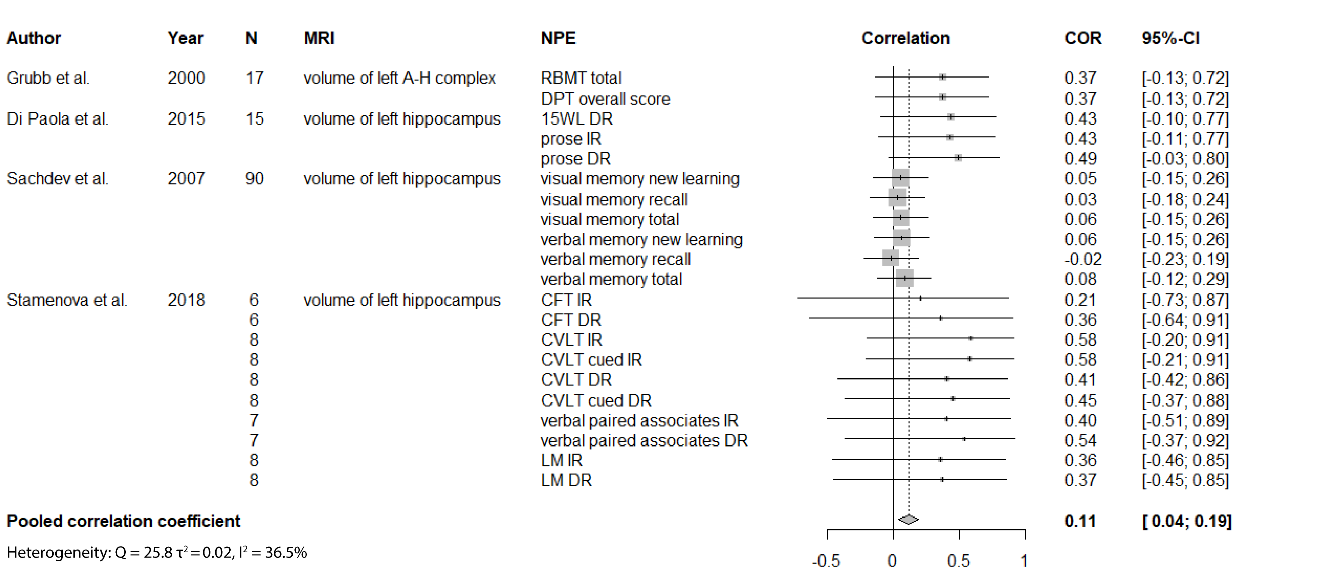


A

*
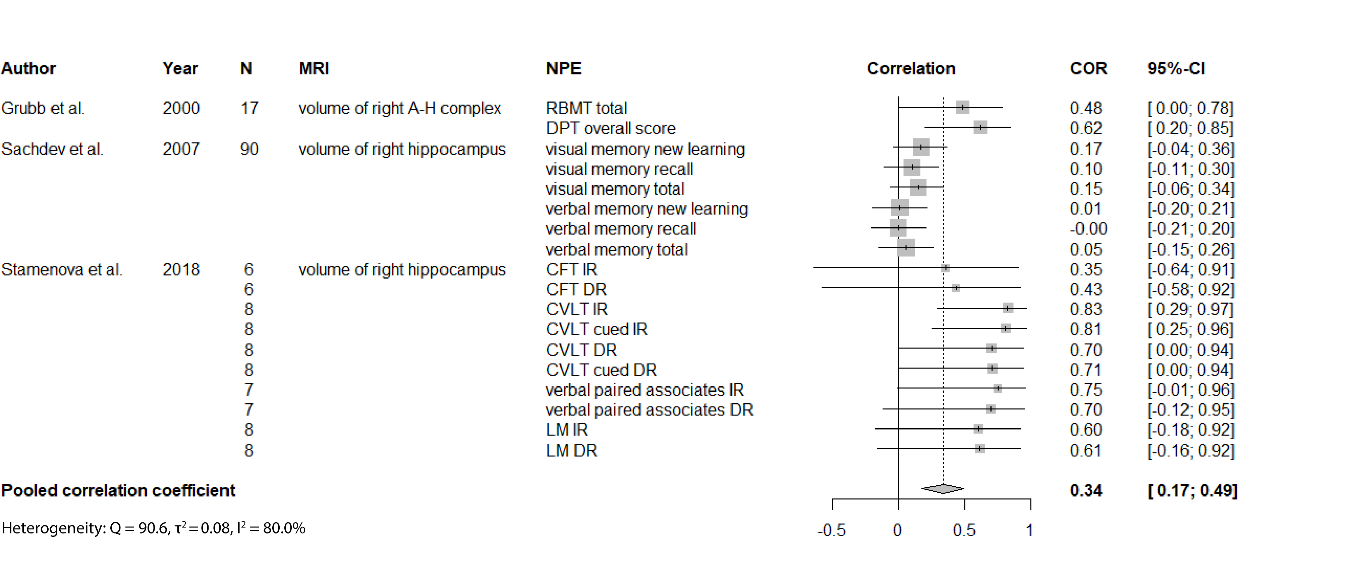
*

*B*

***Fig S1*** *Forest plots for the correlations between MRI markers and memory functioning. A. Correlation between volume of left hippocampus and memory functioning. B. Correlation between volume of right hippocampus and memory functioning.*

*15WL = 15 Word List, CFT = Complex Figure Test, CVLT = California Verbal Learning Test, DPT = Doors and People Test, DR = delayed recall, IR = immediate recall, LM = Logical Memory, RBMT = Rivermead Behavioural Memory Test*

1. Datta, S.G., et al., *Post-concussion syndrome: Correlation of neuropsychological deficits, structural lesions on magnetic resonance imaging and symptoms.* Neurol India, 2009. **57**(5): p. 594-8.

2. Kurca, E., S. Sivak, and P. Kucera, *Impaired cognitive functions in mild traumatic brain injury patients with normal and pathologic magnetic resonance imaging.* Neuroradiology, 2006. **48**(9): p. 661-9.

3. Palacios, E.M., et al., *White matter/gray matter contrast changes in chronic and diffuse traumatic brain injury.* J Neurotrauma, 2013. **30**(23): p. 1991-4.

4. Spitz, G., et al., *Detecting lesions after traumatic brain injury using susceptibility weighted imaging: A comparison with fluid-attenuated inversion recovery and correlation with clinical outcome.* J Neurotrauma, 2013. **30**(24): p. 2038-50.

5. Danet, L., et al., *Thalamic amnesia after infarct: The role of the mammillothalamic tract and mediodorsal nucleus.* Neurology, 2015. **85**(24): p. 2107-15.

6. Diao, Q., et al., *Regional structural impairments outside lesions are associated with verbal short-term memory deficits in chronic subcortical stroke.* Oncotarget, 2017. **8**(19): p. 30900-30907.

7. Exner, C., G. Weniger, and E. Irle, *Implicit and explicit memory after focal thalamic lesions.* Neurology, 2001. **57**(11): p. 2054-63.

8. Leff, A.P., et al., *The left superior temporal gyrus is a shared substrate for auditory short-term memory and speech comprehension: evidence from 210 patients with stroke.* Brain, 2009. **132**(Pt 12): p. 3401-10.

9. Liebermann, D., et al., *A dysexecutive syndrome of the medial thalamus.* Cortex, 2013. **49**(1): p. 40-9.

10. Liu, G., et al., *Regional shape abnormalities in thalamus and verbal memory impairment after subcortical infarction.* Neurorehabil Neural Repair, 2019. **33**(6): p. 476-485.

11. Mandzia, J.L., et al., *Imaging and baseline predictors of cognitive performance in minor ischemic stroke and patients with transient ischemic attack at 90 days.* Stroke, 2016. **47**(3): p. 726-31.

12. Martinaud, O., et al., *Anatomy of executive deficit following ruptured anterior communicating artery aneurysm.* Eur J Neurol, 2009. **16**(5): p. 595-601.

13. Muir, R.T., et al., *Trail making test elucidates neural substrates of specific poststroke executive dysfunctions.* Stroke, 2015. **46**(10): p. 2755-61.

14. Schouten, E.A., et al., *Long-term deficits in episodic memory after ischemic stroke: Evaluation and prediction of verbal and visual memory performance based on lesion characteristics.* J Stroke Cerebrovasc Dis, 2009. **18**(2): p. 128-38.

15. van Rooij, F.G., et al., *Executive function declines in the first 6 months after a transient ischemic attack or transient neurological attack.* Stroke, 2017. **48**(12): p. 3323-3328.

16. Allen, J.S., et al., *Correlations between regional brain volumes and memory performance in anoxia.* J Clin Exp Neuropsychol, 2006. **28**(4): p. 457-76.

17. Di Paola, M., et al., *Hippocampal atrophy is the critical brain change in patients with hypoxic amnesia.* Hippocampus, 2008. **18**(7): p. 719-28.

18. Grubb, N.R., et al., *Memory impairment in out-of-hospital cardiac arrest survivors is associated with global reduction in brain volume, not focal hippocampal injury.* Stroke, 2000. **31**(7): p. 1509-14.

19. Orbo, M.C., et al., *Memory performance, global cerebral volumes and hippocampal subfield volumes in long-term survivors of out-of-hospital cardiac arrest.* Resuscitation, 2018. **126**: p. 21-28.

20. Stamenova, V., et al., *Long-term effects of brief hypoxia due to cardiac arrest: Hippocampal reductions and memory deficits.* Resuscitation, 2018. **126**: p. 65-71.

21. Ariza, M., et al., *Hippocampal head atrophy after traumatic brain injury.* Neuropsychologia, 2006. **44**(10): p. 1956-61.

22. Di Paola, M., et al., *Selective cognitive dysfunction is related to a specific pattern of cerebral damage in persons with severe traumatic brain injury.* J Head Trauma Rehabil, 2015. **30**(6): p. 402-10.

23. Gale, S.D., et al., *Traumatic brain injury and grey matter concentration: A preliminary voxel based morphometry study.* J Neurol Neurosurg Psychiatry, 2005. **76**(7): p. 984-8.

24. Himanen, L., et al., *Cognitive functions in relation to MRI findings 30 years after traumatic brain injury.* Brain Inj, 2005. **19**(2): p. 93-100.

25. Killgore, W.D.S., et al., *Gray matter volume and executive functioning correlate with time since injury following mild traumatic brain injury.* Neurosci Lett, 2016. **612**: p. 238-244.

26. Lauer, J., et al., *Neural correlates of visual memory in patients with diffuse axonal injury.* Brain Inj, 2017. **31**(11): p. 1513-1520.

27. Livny, A., et al., *Cognitive deficits post-traumatic brain injury and their association with injury severity and gray matter volumes.* J Neurotrauma, 2017. **34**(7): p. 1466-1472.

28. Mathias, J.L., et al., *Neuropsychological and information processing performance and its relationship to white matter changes following moderate and severe traumatic brain injury: A preliminary study.* Appl Neuropsychol, 2004. **11**(3): p. 134-52.

29. Merkley, T.L., et al., *Structural and functional changes of the cingulate gyrus following traumatic brain injury: Relation to attention and executive skills.* J Int Neuropsychol Soc, 2013. **19**(8): p. 899-910.

30. Munivenkatappa, A., et al., *Threetime point view of mild brain injuries' structural alteration and their association with cognitive domains.* J Neurosurg Sci, 2019. **63**(5): p. 525-530.

31. Ostberg, A., et al., *Volume change in frontal cholinergic structures after traumatic brain injury and cognitive outcome.* Front Neurol, 2020. **11**: p. 832.

32. Palacios, E.M., et al., *Long-term declarative memory deficits in diffuse TBI: Correlations with cortical thickness, white matter integrity and hippocampal volume.* Cortex, 2013. **49**(3): p. 646-57.

33. Spitz, G., et al., *Regional cortical volume and cognitive functioning following traumatic brain injury.* Brain Cogn, 2013. **83**(1): p. 34-44.

34. Stewan Feltrin, F., et al., *Longitudinal changes in brain volumetry and cognitive functions after moderate and severe diffuse axonal injury.* Brain Inj, 2018. **32**(10): p. 1208-1217.

35. Vannorsdall, T.D., et al., *A morphometric analysis of neuroanatomic abnormalities in traumatic brain injury.* J Neuropsychiatry Clin Neurosci, 2010. **22**(2): p. 173-81.

36. Ware, J.B., et al., *Relationship of cerebral blood flow to cognitive function and recovery in early chronic traumatic brain injury.* J Neurotrauma, 2020. **37**(20): p. 2180-2187.

37. Wright, M.J., et al., *Early metabolic crisis-related brain atrophy and cognition in traumatic brain injury.* Brain Imaging Behav, 2013. **7**(3): p. 307-15.

38. Auriat, A.M., et al., *The impact of covert lacunar infarcts and white matter hyperintensities on cognitive and motor outcomes after stroke.* J Stroke Cerebrovasc Dis, 2019. **28**(2): p. 381-388.

39. Chen, L., et al., *Relationship between hippocampal subfield volumes and memory deficits in patients with thalamus infarction.* Eur Arch Psychiatry Clin Neurosci, 2016. **266**(6): p. 543-55.

40. Jokinen, H., et al., *Medial temporal lobe atrophy and memory deficits in elderly stroke patients.* Eur J Neurol, 2004. **11**(12): p. 825-32.

41. Jokinen, H., et al., *White matter hyperintensities as a predictor of neuropsychological deficits post-stroke.* J Neurol Neurosurg Psychiatry, 2005. **76**(9): p. 1229-33.

42. Munir, M., et al., *Longitudinal brain atrophy rates in transient ischemic attack and minor ischemic stroke patients and cognitive profiles.* Front Neurol, 2019. **10**: p. 18.

43. Sachdev, P.S., et al., *Hippocampal size and dementia in stroke patients: The Sydney stroke study.* J Neurol Sci, 2007. **260**(1-2): p. 71-7.

44. Sachdev, P.S., et al., *Amygdala in stroke/transient ischemic attack patients and its relationship to cognitive impairment and psychopathology: The Sydney Stroke Study.* Am J Geriatr Psychiatry, 2007. **15**(6): p. 487-96.

45. Sachdev, P.S., et al., *Progression of cognitive impairment in stroke/TIA patients over 3 years.* J Neurol Neurosurg Psychiatry, 2014. **85**(12): p. 1324-30.

46. Schaapsmeerders, P., et al., *Ipsilateral hippocampal atrophy is associated with long-term memory dysfunction after ischemic stroke in young adults.* Hum Brain Mapp, 2015. **36**(7): p. 2432-42.

47. Selnes, P., et al., *Hippocampal complex atrophy in poststroke and mild cognitive impairment.* J Cereb Blood Flow Metab, 2015. **35**(11): p. 1729-37.

48. Vataja, R., et al., *MRI correlates of executive dysfunction in patients with ischaemic stroke.* Eur J Neurol, 2003. **10**(6): p. 625-31.

49. Christ, N., V. Mocke, and F. Fluri, *Cerebral microbleeds are associated with cognitive decline early after ischemic stroke.* J Neurol, 2019. **266**(5): p. 1091-1094.

50. Divya, K.P., et al., *Post-stroke cognitive impairment: A cross-sectional comparison study between mild cognitive impairment of vascular and non-vascular etiology.* J Neurol Sci, 2017. **372**: p. 356-362.

51. Gregoire, S.M., et al., *Strictly lobar microbleeds are associated with executive impairment in patients with ischemic stroke or transient ischemic attack.* Stroke, 2013. **44**(5): p. 1267-72.

52. Pohjasvaara, T.I., et al., *White matter lesions are related to impaired instrumental activities of daily living poststroke.* J Stroke Cerebrovasc Dis, 2007. **16**(6): p. 251-8.

53. Tang, W.K., et al., *Absence of cerebral microbleeds predicts reversion of vascular 'cognitive impairment no dementia' in stroke.* Int J Stroke, 2011. **6**(6): p. 498-505.

54. Yatawara, C., et al., *The role of cerebral microbleeds in the incidence of post-stroke dementia.* J Neurol Sci, 2020. **412**: p. 116736.

55. Arenth, P.M., et al., *Corpus callosum integrity and neuropsychological performance after traumatic brain injury: a diffusion tensor imaging study.* J Head Trauma Rehabil, 2014. **29**(2): p. E1-E10.

56. Baek, S.O., et al., *Relation between cingulum injury and cognition in chronic patients with traumatic brain injury; diffusion tensor tractography study.* NeuroRehabilitation, 2013. **33**(3): p. 465-71.

57. Chang, M.C., et al., *The relation between fornix injury and memory impairment in patients with diffuse axonal injury: a diffusion tensor imaging study.* NeuroRehabilitation, 2010. **26**(4): p. 347-53.

58. Chiou, K.S., et al., *Longitudinal examination of the relationship between changes in white matter organization and cognitive outcome in chronic TBI.* Brain Inj, 2019. **33**(7): p. 846-853.

59. Chung, S., et al., *Altered relationship between working memory and brain microstructure after mild traumatic brain injury.* AJNR Am J Neuroradiol, 2019. **40**(9): p. 1438-1444.

60. Dall'Acqua, P., et al., *Functional and structural network recovery after mild traumatic brain injury: A 1-year longitudinal study.* Front Hum Neurosci, 2017. **11**: p. 280.

61. De Simoni, S., et al., *Disconnection between the default mode network and medial temporal lobes in post-traumatic amnesia.* Brain, 2016. **139**(Pt 12): p. 3137-3150.

62. De Simoni, S., et al., *Altered caudate connectivity is associated with executive dysfunction after traumatic brain injury.* Brain, 2018. **141**(1): p. 148-164.

63. Fagerholm, E.D., et al., *Disconnection of network hubs and cognitive impairment after traumatic brain injury.* Brain, 2015. **138**(Pt 6): p. 1696-709.

64. Geary, E.K., et al., *Verbal learning differences in chronic mild traumatic brain injury.* J Int Neuropsychol Soc, 2010. **16**(3): p. 506-16.

65. Grossman, E.J., et al., *Cognitive impairment in mild traumatic brain injury: a longitudinal diffusional kurtosis and perfusion imaging study.* AJNR Am J Neuroradiol, 2013. **34**(5): p. 951-7, S1-3.

66. Gu, L., et al., *Detection of white matter lesions in the acute stage of diffuse axonal injury predicts long-term cognitive impairments: a clinical diffusion tensor imaging study.* J Trauma Acute Care Surg, 2013. **74**(1): p. 242-7.

67. Hellyer, P.J., et al., *Individual prediction of white matter injury following traumatic brain injury.* Ann Neurol, 2013. **73**(4): p. 489-99.

68. Jang, S.H., S.H. Kim, and H. Do Lee, *Relation between memory impairment and the fornix injury in patients with mild traumatic brain injury: A diffusion tensor tractography study.* Am J Phys Med Rehabil, 2018. **97**(12): p. 892-896.

69. Kinnunen, K.M., et al., *White matter damage and cognitive impairment after traumatic brain injury.* Brain, 2011. **134**(Pt 2): p. 449-63.

70. Kondo, K., et al., *The pathophysiology of prospective memory failure after diffuse axonal injury: Lesion-symptom analysis using diffusion tensor imaging.* BMC Neurosci, 2010. **11**: p. 147.

71. Kraus, M.F., et al., *White matter integrity and cognition in chronic traumatic brain injury: A diffusion tensor imaging study.* Brain, 2007. **130**(Pt 10): p. 2508-19.

72. Kuceyeski, A., et al., *The generation and validation of white matter connectivity importance maps.* Neuroimage, 2011. **58**(1): p. 109-21.

73. Munivenkatappa, A., et al., *Role of the thalamus in natural recovery of cognitive impairment in patients with mild traumatic brain injury.* Brain Inj, 2016. **30**(4): p. 388-392.

74. Munivenkatappa, A., et al., *A longitudinal study of changes in Diffusion Tensor Value and their association with cognitive sequelae among patients with mild head injury.* J Neurosurg Sci, 2017. **61**(3): p. 283-290.

75. Owens, J.A., et al., *White matter integrity of the medial forebrain bundle and attention and working memory deficits following traumatic brain injury.* Brain Behav, 2017. **7**(2): p. e00608.

76. Palacios, E.M., et al., *Diffusion tensor imaging differences relate to memory deficits in diffuse traumatic brain injury.* BMC Neurol, 2011. **11**: p. 24.

77. Rabinowitz, A.R., et al., *Neuropsychological recovery trajectories in moderate to severe traumatic brain injury: Influence of patient characteristics and diffuse axonal injury.* J Int Neuropsychol Soc, 2018. **24**(3): p. 237-246.

78. Rajagopalan, V., et al., *Fractal dimension brain morphometry: A novel approach to quantify white matter in traumatic brain injury.* Brain Imaging Behav, 2019. **13**(4): p. 914-924.

79. Shah, S., et al., *Diffusion tensor imaging and volumetric analysis of the ventral striatum in adults with traumatic brain injury.* Brain Inj, 2012. **26**(3): p. 201-10.

80. Solmaz, B., et al., *Assessing connectivity related injury burden in diffuse traumatic brain injury.* Hum Brain Mapp, 2017. **38**(6): p. 2913-2922.

81. Spitz, G., et al., *White matter integrity following traumatic brain injury: The association with severity of injury and cognitive functioning.* Brain Topogr, 2013. **26**(4): p. 648-60.

82. Sugiyama, K., et al., *Clinical utility of diffusion tensor imaging for evaluating patients with diffuse axonal injury and cognitive disorders in the chronic stage.* J Neurotrauma, 2009. **26**(11): p. 1879-90.

83. van der Horn, H.J., et al., *Altered wiring of the human structural connectome in adults with mild traumatic brain injury.* J Neurotrauma, 2017. **34**(5): p. 1035-1044.

84. Wallace, E.J., et al., *Chronic white matter changes detected using diffusion tensor imaging following adult traumatic brain injury and their relationship to cognition.* Neuropsychology, 2020. **34**(8): p. 881-893.

85. Yamagata, B., et al., *Widespread white matter aberrations are associated with phonemic verbal fluency impairment in chronic traumatic brain injury.* J Neurotrauma, 2020. **37**(7): p. 975-981.

86. Yoo, J.S., et al., *Relation between cognition and neural connection from injured cingulum to brainstem cholinergic nuclei in chronic patients with traumatic brain injury.* Brain Inj, 2014. **28**(10): p. 1257-61.

87. Schaapsmeerders, P., et al., *Lower ipsilateral hippocampal integrity after ischemic stroke in young adults: A long-term follow-up study.* PLoS One, 2015. **10**(10): p. e0139772.

88. Zuo, L.J., et al., *The relationship between cerebral white matter integrity and cognitive function in mild stroke with basal ganglia region infarcts.* Sci Rep, 2018. **8**(1): p. 8422.

89. Bernier, R.A., et al., *Dedifferentiation does not account for hyperconnectivity after traumatic brain injury.* Front Neurol, 2017. **8**: p. 297.

90. Palacios, E.M., et al., *Resting-state functional connectivity alterations associated with six-month outcomes in mild traumatic brain injury.* J Neurotrauma, 2017. **34**(8): p. 1546-1557.

91. Santhanam, P., et al., *Effects of mild traumatic brain injury and post-traumatic stress disorder on resting-state default mode network connectivity.* Brain Res, 2019. **1711**: p. 77-82.

92. Shumskaya, E., et al., *Abnormal connectivity in the sensorimotor network predicts attention deficits in traumatic brain injury.* Exp Brain Res, 2017. **235**(3): p. 799-807.

93. Xiong, K.L., et al., *Brain functional connectivity and cognition in mild traumatic brain injury.* Neuroradiology, 2016. **58**(7): p. 733-9.

94. Xu, H., et al., *Longitudinal changes of caudate-based resting state functional connectivity in mild traumatic brain injury.* Front Neurol, 2018. **9**: p. 467.

95. Liu, J., et al., *Altered functional connectivity in patients with post-stroke memory impairment: A resting fMRI study.* Exp Ther Med, 2017. **14**(3): p. 1919-1928.

96. Peng, C.Y., et al., *Regional coherence alterations revealed by resting-state fMRI in post-stroke patients with cognitive dysfunction.* PLoS One, 2016. **11**(7): p. e0159574.

97. Tuladhar, A.M., et al., *Default mode network connectivity in stroke patients.* PLoS One, 2013. **8**(6): p. e66556.

98. Yao, G., et al., *Alterations of functional connectivity in stroke patients with basal ganglia damage and cognitive impairment.* Front Neurol, 2020. **11**: p. 980.
